# Genomic and functional characterization of the L‑sorbose phosphotransferase system in high-risk *Escherichia coli* lineages

**DOI:** 10.1128/msystems.01274-25

**Published:** 2025-12-09

**Authors:** Lena-Sophie Swiatek, Elias Eger, Kristin Surmann, Fynn Meller, Lukas Schulig, Marco Harms, Thaddäus Echelmeyer, Christian Hentschker, Uwe Völker, Michael Schwabe, Katharina Schaufler

**Affiliations:** 1 Department of Epidemiology and Ecology of Antimicrobial Resistance, Helmholtz Institute for One Health, Helmholtz Centre for Infection Research HZI 657809 , Greifswald, Germany; 2 Department of Functional Genomics, Interfaculty Institute for Genetics and Functional Genomics, University Medicine Greifswald 60634, Greifswald, Germany,; 3 University of Greifswald, Institute of Pharmacy, Pharmaceutical and Medicinal Chemistry 26552, Greifswald, Germany; 4 University Medicine Greifswald 60634, Greifswald, Germany; Fluxus Inc., Sunnyvale, California, USA

**Keywords:** *E. coli*, antimicrobial resistance, sorbose and associated fitness, competition, multi-omics investigation

## Abstract

Certain *Escherichia coli* strains belong to international high-risk clonal lineages exhibiting a combination of multidrug resistance, enhanced fitness, and increased virulence, posing major public health challenges. Investigation of characteristics beyond antibiotic resistance genes and classical virulence factors is crucial to gain understanding of common traits among these pathogens. Analysis of 22,267 publicly available *E. coli* genomes from five pathogenic and one commensal sequence type (ST) revealed the L-sorbose phosphotransferase system (PTS ) as a pathogen-enriched marker. Although previously investigated for evolutionary insights, this system has not been functionally assessed within high-risk clonal lineages, as performed in the present study. Our multi-omics results demonstrate that the L-sorbose PTS is induced in pathogenic strains of ST131 and ST648 in the presence of L-sorbose, conferring a competitive advantage compared to a commensal ST10 strain. Transcriptomic analysis revealed co-regulated pathways involving metabolism, flagellar motility, and capsular polysaccharide production, indicating roles in niche adaptation. Despite some strain-specific variations, functional assays with knock-out/knock-in mutants and *Galleria mellonella* infection models confirmed the operon’s impact on competition and fitness.

**IMPORTANCE:**

This study highlights the value of combining large-scale genomic analyses with functional validation for identifying and analyzing the L‑sorbose phosphotransferase system in different *E. coli* high-risk clones. This knowledge may be applied in future studies to address the need for identifying alternative, pathogen-specific targets.

## INTRODUCTION

The *Escherichia coli* species comprises highly diverse strains and includes both commensal representatives, integral to the healthy human intestinal microbiome, and pathogenic types (1). Pathogenic *E. coli* strains are typically classified into intestinal pathogenic *E. coli* (InPEC) and extraintestinal pathogenic *E. coli* (ExPEC) (1, 2). ExPEC colonizes the intestine and can cause a wide range of diseases, including urinary tract infections, sepsis, and meningitis (3, 4). Phylogenetic analysis has separated *E. coli* into phylogroups, with phylogroups B2, D, and F predominating in extraintestinal infections (5).

Certain ExPEC sequence types (STs), such as the globally dominant ST131, ST648, and ST38, have emerged as high-risk clonal lineages that combine multidrug-resistant (MDR) with virulence and fitness traits (6, 7). These clonal lineages, characterized by their high international prevalence, are often equipped with plasmid-encoded resistance mechanisms (8, 9). The overuse and misuse of antibiotics exert strong selection pressure on bacterial communities selecting for resistant strains (10). Infections with resistant strains, especially when combined with high virulence, severely affect patient outcomes (11). The highest number of deaths associated with or attributable to resistance worldwide is caused by resistant isolates of *E. coli* (9).

In contrast, commensal *E. coli* strains, often from phylogroup A, such as ST10, are generally non-pathogenic. However, these strains can harbor antimicrobial resistance (AMR) genes and act as opportunistic pathogens, particularly in immunocompromised individuals (2, 5, 6). The diversity in pathogenic potential across *E. coli* subtypes reflects their large accessory genome, which frequently includes virulence-associated genes and contributes to their adaptability and varied roles in human health (1, 12). However, diversity goes beyond classical resistance and virulence genes (13). Therefore, this study explored the L-sorbose phosphotransferase system (PTS) (*sor*-operon) potentially associated with virulence or fitness in international high-risk clonal lineages of MDR *E. coli*, such as the L-sorbose phosphotransferase system (PTS) (*sor*-operon). Previous research has investigated the evolutionary and diagnostic relevance of L-sorbose utilization in *E. coli* and *Shigella* spp. (*n* = 266), primarily focusing on InPEC (14). That work applied PCR and Sanger sequencing, revealing the presence of the operon encoding an L-sorbose-specific PTS without demonstrating its direct role in conferring a benefit, such as a connection between this metabolic pathway and the fitness or virulence in MDR high-risk clonal lineages (14). Through homologous clustering of 22,267 publicly available *E. coli* genomes, we identified the L-sorbose PTS enriched in high-risk clonal lineages. Detailed multi-omics analysis of two strains from ST131 and ST648 revealed the *sor*-operon as a determinant of fitness and competitive advantage, which is linked to co-regulated pathways such as flagellar motility and capsular polysaccharide production. Its involvement in these pathways was further validated through knock-out and knock-in experiments.

## MATERIALS AND METHODS

### Bacterial strains

Homologous clustering included six STs: ST10 as a commensal representative (phylogroup A), often isolated as a colonizer, and ST38 (phylogroup D), ST131 (phylogroup B2), ST405 (phylogroup D), ST410 (phylogroup C), and ST648 (phylogroup F) as pathogenic representatives based on their global success as high-risk clonal lineages (6). Initial microbiological screenings were conducted for three strains of each ST (Table S1). We included two mutants of the wild-type strain PBIO729 (ST131): PBIO729∆*sorAM*, with a deletion in transport component genes, and PBIO729∆*sorE*, which cannot reduce L-sorbose-1-phosphate to d sorbitol-6-phosphate. Additionally, a *sor-*operon knock-in strain (PBIO365:*sor*, ST10) with insertion into the region identical to *sor* positive strains was included. For in-depth analysis, including transcriptomics, proteomics, and phenotypic characterization, we selected one strain from each of ST10 (PBIO365), ST131 (PBIO729), and ST648 (PBIO730). ST131 was chosen as the dominant ExPEC lineage, while ST648 was selected due to its emergence in multiple regions as a potential pandemic clone (6, 15–17). Both combine epidemiological relevance with complementary biological traits and differ in phylogroups and certain phenotypic features. Within each selected ST, we chose isolates from our in-house collection that are extensively characterized with phenotypic data and high-quality whole-genome sequences, enabling robust multi-omics integration (16, 18, 19).

### Data set and homologous clustering

*E. coli* genomes (Table S1) were downloaded from EnteroBase database (v1.2.0) (20) (*n* = 24,724; Dec. 2020) and filtered (L50-value ≥ 20 contigs), allowing 5% missing genes within the Benchmarking Universal Single-Copy Orthologue (BUSCO, v 5.0) and the *Enterobacterales* odb10 analysis (21). Twenty-eight genomes, either publicly available via NCBI or provided by cooperation partners, were integrated to the data set. Reads were quality trimmed with Trim Galore (v. 0.6.8; https://github.com/FelixKrueger/TrimGalore) and assembled with Shovill (v. 1.1.0; https://github.com/tseemann/shovill), if only raw data were available. Genome assemblies were annotated using Prokka (v. 1.14.6) (22) and homologous clustering using cd-hit (v. 4.8.1) (23) with protein sequences as input (cut-offs: 95% identity and coverage). Pathogen-enriched markers were characterized by their presence in ≥90% of each ST of the pathogenic genomes and in ≤10% of the commensal genomes. BLAST analysis was performed using the non-redundant databases of the National Center for Biotechnology Information (24) and UniProt (25). To examine potential bias from artificial laboratory strains (e.g., *E. coli* K-12 MG1655), *E. coli* genomes belonging to ST10 were filtered based on the “Name” column in EnteroBase. If a name (e.g., *E. coli* K-12 MG1655) occurred ≥10 times, one assembly was randomly chosen as a representative.

### Cultivation and harvest of bacterial strains

Bacterial strains (including PBIO365, PBIO2, PBIO729, PBIO730, PBIO729∆*sorAM*, PBIO729∆*sorE*, PBIO365:*sor*) were cultivated in Nutrient Broth II (NB) without (ctrl) or with 1% L-sorbose (sor) (Carl Roth, Karlsruhe, Germany), shaking at 150–220 rpm, 37°C, unless stated otherwise (Table S1). For growth kinetics, 5 mL of NB (ctrl or sor), LB, or M9 minimal medium were inoculated with a single colony of the respective strain and incubated overnight. Either 10 mL of medium was inoculated at an optical density of 0.05 at *λ* = 600 nm (OD_600_), and growth was monitored by measurements every 30 min for 8 h, or 200 µL was transferred into a 96-well plate, and growth was assessed automatically every 30 min for 24 h using a plate reader (CLARIOstarplus, BMG Labtech, Ortenberg, Germany).

Total protein and RNA were extracted from the same cultures (PBIO365, PBIO720, and PBIO730), which were initiated through a systematic serial dilution of 5 mL NB overnight cultures originating from a glycerol stock. Pre-cultures (20 mL of NB) were inoculated starting from mid-exponential phase overnight cultures (referring to an OD_600_ of approx. 0.6-1.0), and main cultures (80 mL NB ctrl or sor) were initiated from mid-exponential phase pre-cultures at an OD_600_ of 0.05. Cell harvest was performed during the early stationary growth phase and involved cooling in liquid nitrogen and centrifugation (3 min; 4°C; 8,000 × *g*). Cell pellets were stored at −80°C until RNA and protein preparation.

### Visualization of L-sorbose utilization in pathogenic *E. coli* strains

The utilization of L-sorbose was evaluated as described previously using NB-agar plates supplemented with 1% L-sorbose and 50 mg/L neutral red (14). Utilization of other sugars, including D-sorbitol (Merck), D-glucose (Carl Roth), D-fructose (Merck), and D-mannose (Sigma Aldrich; Merck), was equally tested.

### Isolation of total DNA

Total DNA extraction was performed using the MasterPure DNA Purification Kit for Blood, Version 2 (Lucigen, Middleton, WI, USA), according to the manufacturer’s instructions. DNA was quantified with dsDNA HS Assay Kit using a Qubit 4 fluorometer (Thermo Fisher Scientific, Waltham, MA, USA) (e.g., 21).

### Mutant generation and sequencing

Marker-less mutant strains PBIO729∆*sorAM*, PBIO729∆*sorE*, and PBIO365:*sor* were generated by Creative Biogene (Shirley, NY, USA) through homologous recombination. Chromosomal DNA of the mutants was isolated and then shipped to SeqCenter (Pittsburgh, PA, USA) (Supplementary methods I).

### Preparation of total RNA

For transcriptomic analysis and Northern blotting, total bacterial RNA was prepared by mechanical disruption and acid phenol-chloroform extraction as previously described, with minor modifications (26). Modifications concerned the resuspension upon mechanical disruption in 2 mL only, as a bacterial pellet equivalent to 8 OD units was used (see Table S2 for the composition of all buffers).

### Northern blot analysis

The *sor*-operon expression levels were analyzed by using *sorC* or *sorE* digoxigenin-labeled probes (Supplementary methods II).

### Database for omics analysis

To compare the expression and translation profiles in the presence and absence of L-sorbose between PBIO365, PBIO729, and PBIO730, a pangenome was built with PIRATE (v. 1.0.4, -s 95,96,97,98,99,100, -k -cd-low 100 -e 1E-12 -hsp-len 0.5). This resulted in a grouping of genes from all three strains and the assignment of a unique group ID. The resulting representative protein sequences were used as input for proteomic analysis to minimize redundancy and enable consistent peptide mapping across strains.

### RNA sequencing and differential gene expression analysis

Total RNA samples of PBIO365, PBIO729, and PBIO730 were frozen and shipped to LGC Genomics GmbH (Berlin, Germany). The rRNA depletion and mRNA library preparation was performed by LGC Genomics GmbH (Berlin, Germany), and sequencing was conducted with 1 × 75 bp reads (Illumina NextSeq 550, non-stranded). Raw reads were adapter- and quality-trimmed using Trim Galore. Trimmed reads were mapped using Bowtie 2 (v. 2.4.5/mode: -very-sensitive-local) (27) with the genome assemblies as references for the individual strains. Gene counts were calculated using featureCounts (v. 2.0.1) (28) based on the annotation for each strain. The count table was imported into RStudio (v. 4.3.1). Differentially expressed genes were identified with DESeq2 (v. 1.40.0) in default mode by comparing counts from RNA preparations in the absence and presence of L-sorbose. Log2 fold-change (L2FC) was calculated by comparing individual strains grown in the presence and absence of L-sorbose.

### Isolation of bacterial proteins

The bacterial cell pellets were processed as described before (29) and washed twice (4°C, 10 min, 17,000 × *g*) with ice-cold phosphate-buffered saline (PBS; Thermo Fisher Scientific). The pellets were then reconstituted in 100 µL of 20 mM 4-(2-hydroxyethyl)-1-piperazineethanesulfonic acid (HEPES; pH 8.0) with 2% (wt/vol) sodium dodecyl sulfate (SDS), followed by denaturation (95°C, 1 min) with vigorous shaking. Cell disruption was achieved using pre-cooled vessels and a Mixer Mill MM 400 (Retch, Haan, Germany) for 3 min at 2,600 rpm. Cell powder was resuspended in 150 µL of preheated (95°C) 20 mM HEPES (pH 8.0) and transferred into a 1.5 mL low binding pre-lubricated tube (Sorenson BioScience, Salt Lake City, UT, USA). After cooling to room temperature (RT), lysates were supplemented with 4 mM MgCl_2_ and 0.005 U/µL benzonase (Pierce Universal Nuclease, Thermo Fisher Scientific). Ultrasonication was carried out for 5 min, and cell debris was pelleted by centrifugation (30 min; 17,000 × *g*; RT). The resulting supernatants were then transferred into a fresh 1.5 mL low binding pre-lubricated tube (SorensonTM BioScience).

### Bicinchoninic acid assay

Protein concentration was determined as described previously (29, 30), using the MicroBCA Protein Assay Kit (Thermo Fisher Scientific), according to the manufacturer’s instructions. Measurements were conducted using BioTek Synergy (Agilent Technologies, Waldbronn, Germany). The obtained data were analyzed through the Shiny application, as described by Reder et al., to ensure accurate quantification (30).

### Single-plot solid-phase enhanced sample preparation

The single-plot solid-phase enhanced sample preparation, with minor adjustments as described by Blankenburg et al., was executed (29, 31). For trypsin digestion, 5 µg of total protein in 10 µL of 20 mM HEPES (pH 8.0) were incubated (18 min shaking at 1,400 rpm) with 10 µL of magnetic beads, comprising equal volumes of hydrophilic (Speedbead magnetic carboxylated modified particles, GE Healthcare, United Kingdom) and hydrophobic beads (Sera-Mag Speedbead carboxylated-modified particles, Thermo Fisher Scientific). The supernatant was discarded by bead sedimentation on a magnetic rack, followed by two washes with 70% (vol/vol) ethanol and one wash with 100% (vol/vol) acetonitrile (ACN) (Thermo Fisher Scientific) before air-drying. Trypsin digestion occurred in freshly prepared 20 mM ammonium bicarbonate buffer, with a trypsin-to-protein ratio of 1:25 (18 h at 37°C). The digest was stopped by adding ACN, and the protocol was performed as described before (31).

### Acquisition and analysis of mass spectrometry data

Peptides were separated using an UltiMate 3000 nanoLC device (Thermo Fisher Scientific) with a pre-column (Acclaim PepMap; Thermo Fisher Scientific) and an analytical column (Accucore; Thermo Fisher Scientific) by applying a binary gradient with buffer A (0.1% [vol/vol] acetic acid in HPLC-grade water) and buffer B (0.1% [vol/vol] acetic acid in ACN) at a flow rate of 300 nL/min, as described previously (29). After ionization, peptides were analyzed with a Q Exactive HF mass spectrometer (Thermo Fisher Scientific) in data-independent acquisition mode. Specification of the used gradient and detailed settings on nanoLC-MS/MS data acquisition are provided (Table S5). Raw data were mapped against the in-house established database (see 2.9), including whole-genome sequences of analyzed strains, using Spectronaut (v. 16.0) (Biognosys, Schlieren, Switzerland). All search parameters are provided (Supplementary methods III, Table S6). Statistical comparison was accomplished on peptide level with the PECA package (v. 1.30.0) by applying a ROTS test (ROPECA approach—reproducibility-optimized peptide change averaging) (32). Protein intensities were calculated using the maxLFQ algorithm (33). Proteome data have been stored at the ProteomeXchange Consortium via the PRIDE partner repository (34) with the dataset identifier PXD056542.

### Multi-omics marker analysis

The multi-omics analysis was performed using three biological replicates for RNA-Seq and four biological replicates for mass spectrometry for each condition and strain. Differential expression was defined as L2FC > 1 or < −1 (|L2FC| > 1.0) with an adjusted *P* ≤ 0.05. Genes not meeting these criteria were considered to exhibit no differential expression. The pathogen-enriched markers included genes that were up- or downregulated in the pathogenic strains (PBIO729 and PBIO730) under sor compared to ctrl (sorbose-free) conditions, but showed no differential expression in the commensal representative (PBIO365). Additionally, we analyzed strain-specific up- or downregulated features. For a multi-step examination, up- or downregulated features were investigated by integrating eggNOG (v. 2.1.9) annotations (35, 36) and the Kyoto Encyclopedia of Genes and Genomes (KEGG) (37). Information regarding the cluster of orthologous groups (COG) was plotted for genes showing up- or downregulation on transcript levels.

### Competition assay

Competition assays assessed the fitness of each strain relative to another strain. Main cultures (NB sor or ctrl) of the respective strain combination were inoculated at a total OD_600_ of 0.05, with a strain ratio of 1:3. Samples were collected at 0 h and 24 h, diluted, and plated on selective agar for differentiation of strains: either LB and LB-cefotaxime (4 µg/mL) (Thermo Fisher Scientific), LB-chloramphenicol (8 µg/mL) (Sigma Aldrich; Merck), or NB agar supplemented with 1% L-sorbose and 50 mg/L neutral red (Merck, Darmstadt, Germany). Differentiation was verified based on colony PCR detecting an amplicon of the *sor-*operon (Table S7).

### Survival in human serum

The survival of the different bacterial strains in 50% human serum was assessed as described previously with minor modifications (38). Strains were incubated in NB without or with 1% L-sorbose overnight (37°C, shaking). Then, bacteria were washed and resuspended in PBS containing either 2% L-sorbose or 2% D-glucose to a final OD_600_ of 0.1. Bacterial suspensions were then mixed with equal amounts of human serum (US origin, Sigma-Aldrich, St. Louis, USA) and seeded in a 96-well plate. Determination of CFU/mL was performed immediately and after 24 h. Serum resistance was expressed as the L2FC in CFU/mL from t_0_ to t_24_.

### Motility assay

Strains were incubated in either NB without or with 1% L-sorbose overnight (37°C, shaking) before diluting cultures 1:100 in fresh medium. Motility agar was prepared using NB with 0.3% agar (Carl Roth), with or without L-sorbose. Before spotting 10 µL of each culture onto agar plates in triplicates, bacterial cells were washed with PBS and adjusted to OD_600_ of 0.5. Plates were incubated at 37°C for 24 h. Motility was determined by measuring the radius of the colony and the swarming.

### Biolog plates

Biolog plates PM1, PM2, PM3, and PM5 (Biolog, Hayward, CA, USA) were used to screen the strains PBIO365, PBIO729, PBIO729∆*sorE*, and PBIO730, following the manufacturer’s protocol with slight modifications (Supplementary methods IV).

### *Galleria mellonella* infection model

For the *Galleria mellonella* infection model, overnight cultures were diluted 1:50 in 20 mL NB and incubated. Then, 1 mL of culture was collected (5 min, 8,000 × *g*) and washed twice with PBS. Optical density was set to OD_600_ of 0.1 (~10^5^ CFU). The larvae of the greater wax moth (*Galleria mellonella*) were used as an *in vivo* infection model, as described previously (39).

### Statistical analysis

All phenotypic experiments were conducted with three or more independent biological replicates, and statistical analyses were performed using GraphPad Prism (v. 9.3.0) for Windows (GraphPad Software, San Diego, CA, USA). Data were expressed as mean and standard deviation unless stated otherwise. Statistical significance was assessed via one-way analysis of variance (ANOVA) with uncorrected Fisher’s LSD, unpaired t with Welch’s correction, or two-way ANOVA with Tukey correction for multiple comparisons. Significance levels were indicated as follows: *P < 0.033; **P < 0.002; ***P < 0.001. For transcriptomic and proteomic analyses, an L2FC greater than 1 (|L2FC| > 1.0) with an adjusted *P*-value equal to or smaller than 0.05 was considered significant.

## RESULTS

### Discovery of the L-sorbose phosphotransferase system as a unique marker in pathogenic *E. coli* lineages

**Fig 1:**
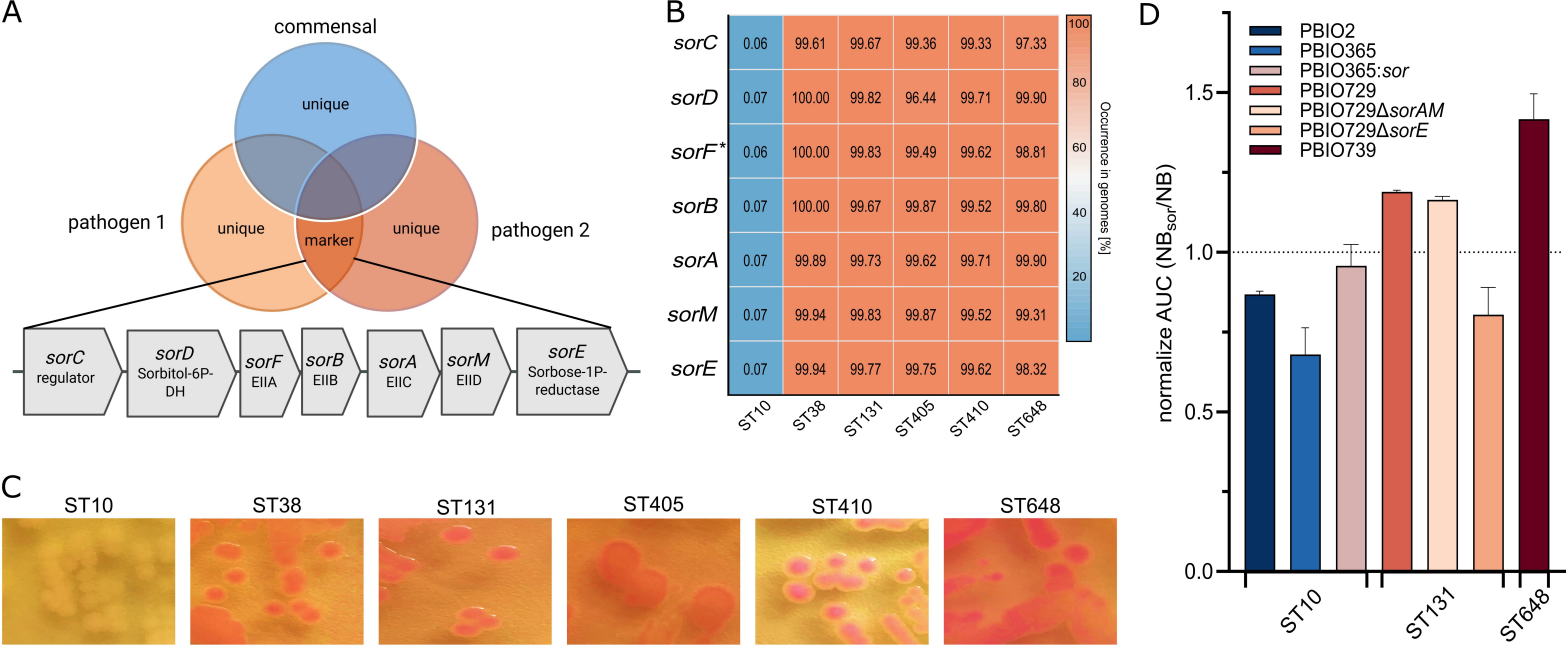
Differences in the occurrence of *sor*-operon reflect in unique L-sorbose utilization in pathogenic STs. (**A**) Venn diagram schematically illustrating the genomic intersections, including the genomic markers and structure of *sor*-operon. Each circle represents the genome of an exemplary strain. Overlapping regions indicate shared genes. The intersection among all pathogenic strains is referred to as marker section, including the *sor*-operon. (**B**) Heatmap of percentage occurrence of the indicated genes of the *sor*-operon. The occurrence in the commensal ST10 and the pathogenic STs (ST38, ST131, ST405, ST410, and ST648) is color coded from blue (0%) to red (100%) as a heatmap. *Clustering of genes was performed with 95% identity and coverage, except for *sorF*, where clustering based on 75% identity is shown because otherwise, genes will occur in separate clusters. (**C**) Single colonies of representative pathogenic and commensal *E. coli* STs on Nutrient Broth II agar containing 1% L-sorbose and neutral red. (**D**) Growth (OD_600 nm_) of pathogenic and commensal *E. coli* STs and the indicated mutants was recorded in the presence and absence of L-sorbose: The growth of each strain in NB and NB*sor* was compared by calculating the ratio of AUC of three independent replicates. These ratios and their respective mean values are presented for the individual strains. Additionally, the respective ST is indicated below.

For homologous clustering of 22,267 *E. coli* genomes, we included six STs: ST10, often isolated in colonization and surveillance studies comprising a high diversity of isolates, and ST38, ST131, ST405, ST410, and ST648, which are globally successful high-risk clonal lineages. For consistency throughout the manuscript, we use “commensal” to refer to ST10 and “pathogenic” for the international high-risk clonal lineages. However, it is important to acknowledge that ST10 has been previously detected in extraintestinal infections, indicating its potential to act as an opportunistic pathogen (6, 7). Most significantly, the homologous clustering detected seven genes from an operon encoding the L-sorbose-specific PTS, which were enriched in the pathogenic STs and largely absent in ST10 (Fig. 1A; Table S8). The operon was previously explored as an evolutionary marker, i.e., a genetic feature indicating phylogenetic relationships among *E. coli* pathotypes and *Shigella* (14). The *sor-*operon genes (*sorC*, *sorD*, *sorB*, *sorA*, *sorM*, and *sorE*) were present in more than 95% of all pathogenic representatives and in less than 0.1% (*n* = 6) of the commensal genomes (Fig. 1B). Four distinct *sorF* clusters were identified when clustered at 95% sequence identity and length, revealing only minor sequence variations. As a result, they were pooled using a 75% sequence identity and length threshold. Further analysis of 44,208 genomes (Supplementary methods V) across all phylogroups revealed that this operon is present in the majority (>86%) of genomes, except for phylogroups A (9.56%) and B1 (12.12%) (Table S9). Investigation of the surrounding regions revealed that, while genes are generally conserved across and within STs, including ST10, there are exceptions and ST-specific insertions (e.g., ST648) (Fig. S1), warranting further investigation.

Genomic analyses suggested potential differences in L-sorbose utilization between ST10 and the other STs. To investigate this, we tested L-sorbose utilization in three strains from each ST on NB L-sorbose agar supplemented with neutral red dye (Fig. S2A; Fig. 1C). Utilization of other sugars (D-sorbitol, D-glucose, D-fructose, and D-mannose) did not reveal any pathogen-specific patterns (Fig. S2B). To examine the impact on growth behavior, we selected two commensal ST10 strains (PBIO365 and PBIO2) and two pathogenic representatives from ST131 (PBIO729) and ST648 (PBIO730). Additionally, a *sor-*operon knock-in mutant (PBIO365:*sor*) and two different mutant strains of PBIO729 lacking either *sorAM* or *sorE* were tested, all exhibiting similar growth kinetics in LB medium and minimal medium supplemented with 1% dglucose (Fig. S3A and B). In minimal medium containing 1% L-sorbose, only PBIO729, PBIO729∆*sorAM,* and PBIO730 grew (Fig. S3C). PBIO365:*sor* grew in minimal medium containing 1% L-sorbose; however, evident growth was observed after 48 h. In complex medium supplemented with 1% L-sorbose (NB*sor*), pathogenic strains and PBIO729∆*sorAM*, but not PBIO729∆*sorE,* displayed growth with cellular densities increasing concurrently with medium acidification during the transient growth phase, indicating the initiation of L-sorbose utilization (Fig. S3D and E). This is reflected in an increased area under the curve (AUC) calculated from the growth kinetics in NB*sor* compared to NB (Fig. 1D). Enhanced growth was also observed when comparing PBIO365 with the integrated *sor*-operon to the wild-type strain. However, PBIO365 exhibited slightly enhanced growth in NB compared to NB*sor*, resulting in a normalized AUC below 1, likely because the inability to utilize L-sorbose disturbs growth in NB*sor* (Fig. 1D). This may result from uptake of L-sorbose via low-specificity PTS transport systems, leading to intracellular accumulation of phosphorylated intermediates that impose osmotic stress and disrupt phosphate homeostasis (40, 41). Notably, PBIO729∆*sorE* exhibited the strongest impairment in growth compared to the wild-type and in relation to growth in NB. We conclude that while the mutant deficient for the transport components, PBIO729∆*sorAM*, is still capable of L-sorbose uptake via other PTSs, the PBIO729∆*sorE* mutant, despite having an intact transport, is unable to reduce L-sorbose-1-phosphate to D-sorbitol-6-phosphate.

### L-sorbose utilization is triggered during early stationary phase among other pathways

Expression was examined for the regulator *sorC,* which is an activator in the presence of L-sorbose and a repressor in the absence, and for sorbose-1-phosphate reductase *sorE* (42). This revealed *sor-*operon induction in the presence of L-sorbose in pathogenic strains during the transition into the stationary growth phase (*sorC*) and during stationary phase (*sorE*), respectively (Fig. S4B). Comparing early stationary growth phase samples in the presence and absence of L-sorbose, we confirmed induction of the *sor*-operon by both transcriptome and proteome analyses in pathogenic strains (PBIO729 and PBIO730), while no induction was detected in the commensal strain PBIO365, as expected due to gene absence (Fig. S4C). Ranking the genes revealed that the seven *sor-*genes exhibited the highest L2FC between the two conditions among all genes detected as differentially expressed. Expression of a shikimate dehydrogenase-like gene (*sdhL*), located immediately upstream of the *sor*-operon, was observed only in the ST648 strain, and *sdhL* is absent from ST131 and the other investigated STs (Fig. S1).

To gain deeper insights into the functional benefits of expressing the L-sorbose PTS and fermenting L-sorbose as an additional carbon source, the overall expression patterns were analyzed to elucidate the associated regulatory pathways. Proteomic analysis confirmed the production of proteins of the *sor*-operon; however, reconstructing a meaningful network of regulatory pathways was not feasible due to the limited number of proteins differently abundant in ST131 and ST648 but not in ST10. Proteomics revealed significant downregulation in metabolic pathways, including propionate metabolism, fatty acid oxidation, and chemotaxis, likely attributable to a shift in cellular resources toward the L-sorbose PTS components ( Table S10, Fig. S5). Transcriptomic analysis identified regulatory pathways shared among all three strains, along with strain-specific induction of gene expression. Comparison of sorbose and control conditions revealed a subset of 127 genes that were upregulated and 30 genes that were downregulated in both pathogenic strains (PBIO729, PBIO730), whereas these were not detected as up- or downregulated genes in the commensal strain (PBIO365) (Fig. S6A). Most up- or downregulated features in the presence of L-sorbose were detected in PBIO729, while the lowest number of genes and proteins was detected in PBIO365 (Fig. S6B). Subsequent analysis indicated that only a small proportion of strain-specific regulatory effects were directly attributable to differences in gene presence. Instead, it appeared that many of these differences occurred due to variations in the regulatory pathways upon l-sorbose utilization (Fig. S6C). Although both PBIO729 (ST131) and PBIO730 (ST648) are pathogenic ExPEC lineages, differences in accessory genome content and regulatory network architecture account for different transcriptional reprogramming.

### Pathogen-specific regulatory networks activated by L-sorbose fermentation

**Fig 2:**
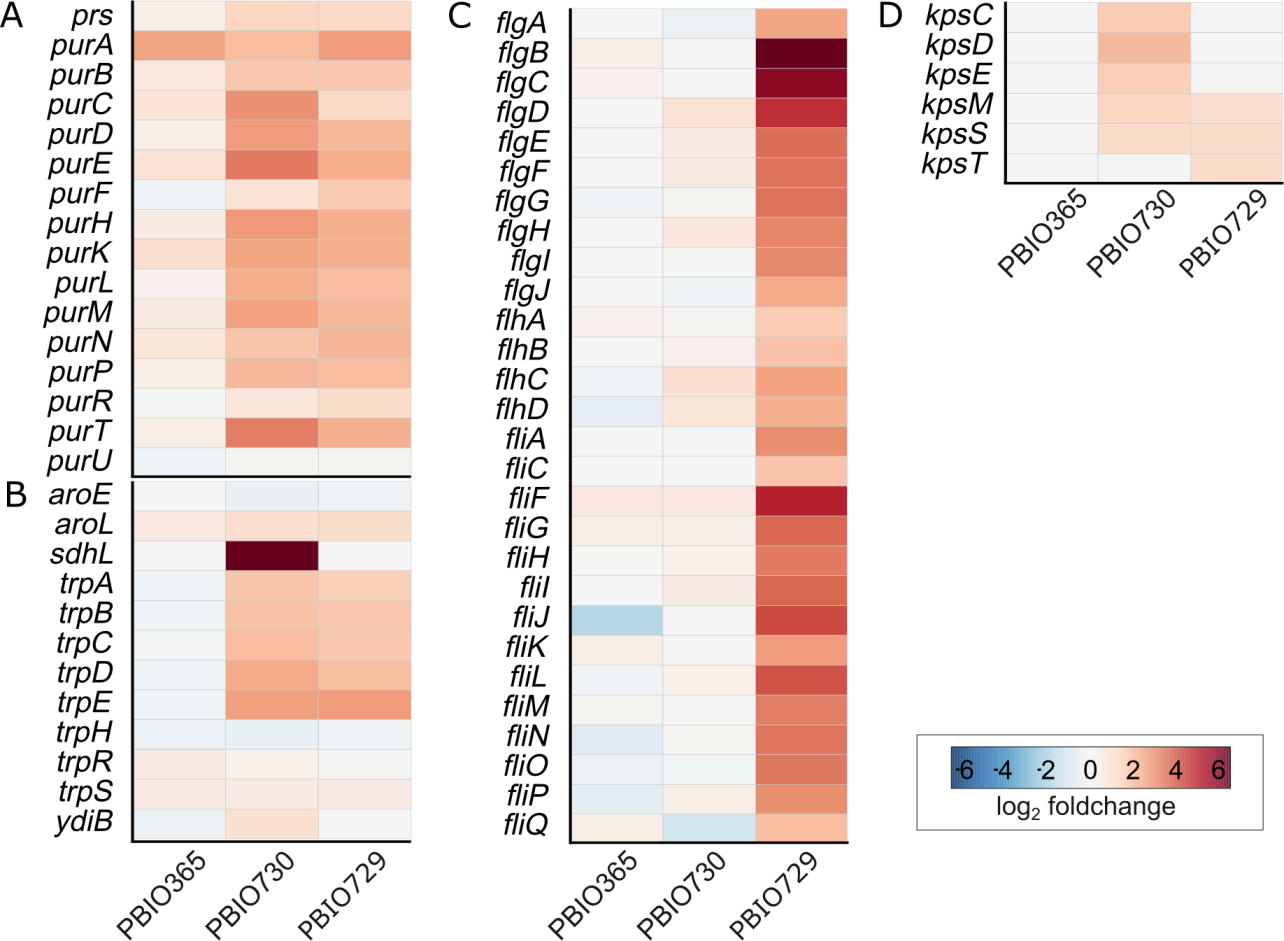
Heatmaps of regulated genes mapping to different virulence- and/or fitness-associated pathways. Based on research using the KEGG database, differentially expressed genes were mapped to their respective pathways, including (**A**) tryptophan metabolism (KEGG-map00380), (**B**) purine metabolism (KEGG-map00230), (**C**) flagellar assembly (KEGG-T30583_02040), and (**D**) *kps*-operon. Heatmaps indicating the differential gene expression of the respective genes (upregulated (L2FC ≥ 1.0; *P* adjusted ≤ 0.05) [red], not differentially expressed [white], downregulated (L2FC ≤ −1.0; *P* adjusted ≤ 0.05) [blue]) for the analyzed strains (PBIO365, PBIO729, PBIO730).

The differentially expressed genes were further analyzed to identify their associated pathways and to better understand the potential impact of these changes. COG analysis revealed an increase and diversification of regulated pathways in pathogenic strains (Fig. S7). We identified two major pathways related to tryptophan and purine metabolism that were upregulated in both pathogenic, but not the commensal strain, in the presence of L-sorbose (Fig. 2). Six genes encoding proteins involved in the tryptophan biosynthesis are organized in an operon-like structure (*trpA/B/C/G/D/E*) in all three strains but are only differentially expressed in the pathogenic representatives (Fig. 2A). Moreover, we identified upregulated genes associated with the shikimate pathway. While *aroL* was upregulated in both pathogenic strains, the *sdhL* gene was differentially expressed in only PBIO730, in accordance with its unique occurrence in ST648, as described above. Purine-associated genes, such as *purA*, were similarly regulated in all three strains. However, genes particularly important for initial steps in this pathway were upregulated in the pathogenic strains, including *purB/C/D/E/F/H/K/L/M/N/P/R/T* (Fig. 2B). The *pur* genes were non-contiguously distributed across multiple chromosomal loci in all three strains, rather than being organized in a single operon, which influences their transcriptional regulation and purine metabolism (43). Diverse upregulation of genes concerning capsule polysaccharide (CPS) production was detected with L-sorbose utilization in pathogenic STs, such as the *kps*-operon (Fig. 2D). In both STs, genes encoded in region 1 and region 3 of the *kps*-operon were upregulated during L-sorbose fermentation (44). Strain-specific responses most prominently included the upregulation of a total of 32 flagellar genes in PBIO729 (Fig. 2C).

### Alignment between transcriptomic changes and observed phenotypic variations

To complement the transcriptomic analysis, selected phenotypic assays using knock-out and knock-in mutants were performed based on the detected upregulated genes, including tryptophan and purine metabolism, flagellar assembly, and capsule polysaccharide synthesis. A Biolog plate-based screening supported the observation of a general enhancement of previously described metabolic pathways (Table S11). Amino acid and peptide metabolism, including L-serine, L-isoleucine, L-leucine, L-lysine, L-methionine, glycyl-L-aspartic acid, and glycyl-L-glutamic acid, showed alterations when comparing PBIO729 under sor versus control conditions and were not detected in PBIO365 or PBIO729∆*sorE.* Enhanced purine metabolism, specifically guanosine, was detected in PBIO729 via Biolog screening, consistent with transcriptomic data showing upregulation of genes for purine metabolism.

**Fig 3:**
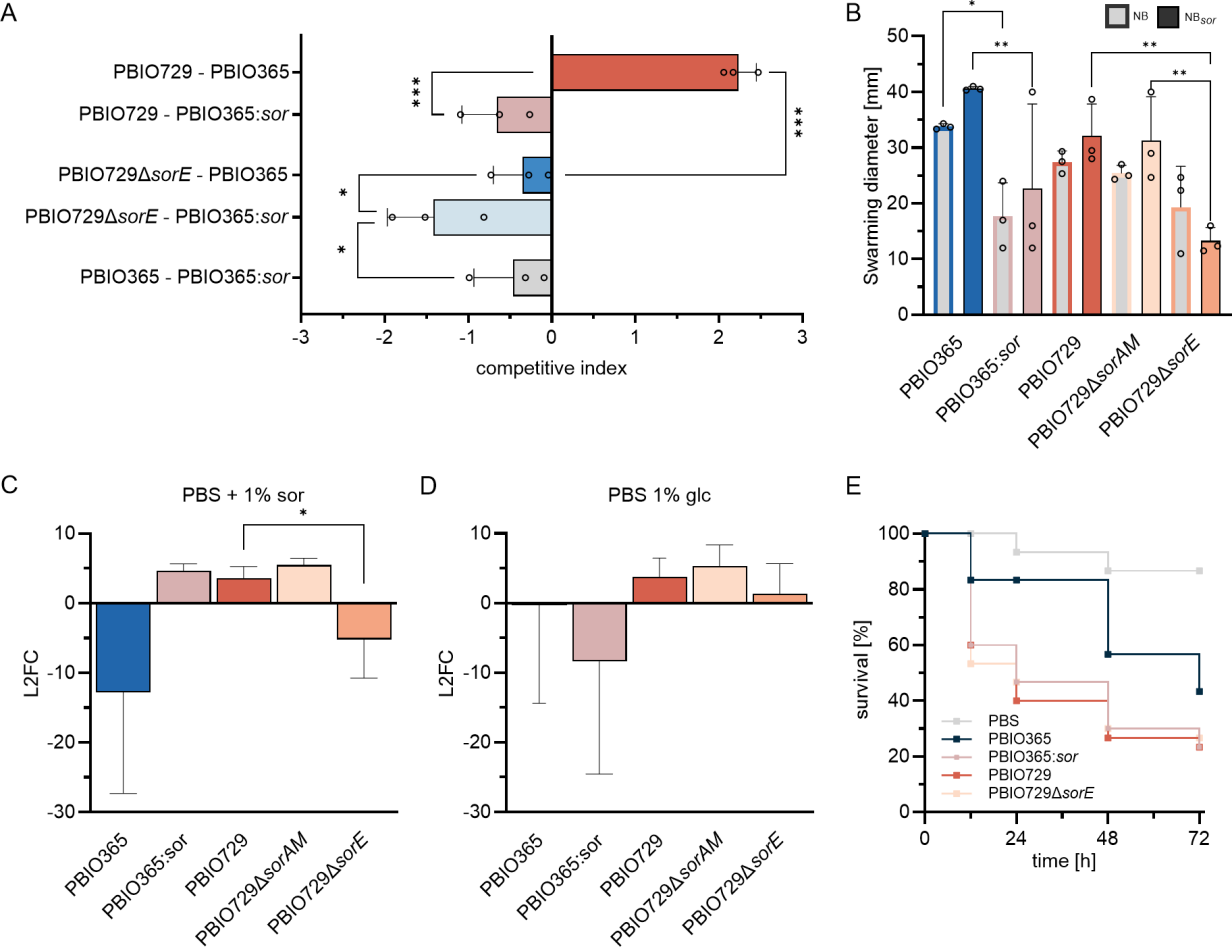
Phenotypic analysis revealed L-sorbose-dependent changes in fitness and/or virulence. The indicated strains were tested in biological replicates (*n* ≥ 3). (**A**) Competitive benefits in nutrient broth medium supplemented with 1% L-sorbose were calculated from the CFU/mL of the individual strains comparing the ratio of test over reference strain after 24 h of competition with the inoculation ratio. Relevant changes were evaluated regarding their significance (ordinary one-way ANOVA with Fisher’s LSD test; adj. *P* < 0.033 [*]; <0.002 [**]; <0.0002 [***]; <0.0001 [****]). (**B**) Motility of strains was tested using NB agar plates (0.3% agar) either without or with 1% L-sorbose, and the radius of swarming is plotted. Relevant changes were evaluated regarding their significance (two-way ANOVA with Tukey correction for multiple comparisons) (adj. *P* < 0.033 [*]; <0.002 [**]; <0.001 [***]). (**C**) Survival was assessed in 50% human serum diluted in PBS with 1% l-sorbose (**D**) or 1% D-glucose. The L2FC was calculated from the inoculum and the CFU/mL after 24 h of incubation. Relevant changes were evaluated regarding their significance (one-way ANOVA, unpaired *t-*test with Welch’s correction; adj. *P* < 0.033 [*]; <0.002 [**]; <0.001 [***]). (**E**) *Galleria mellonella* larvae were infected with the indicated strains using 10 technical and three biological replicates each and PBS as injection control. Survival was assessed every 12–24 h for 72 h and the mean survival rate is plotted.

To evaluate whether L-sorbose utilization provides an additional competitive fitness advantage, the competitive index (CI) was assessed in mixed bacterial cultures (Fig. 3A). The wild-type pathogenic strain PBIO729 demonstrated a clear competitive advantage over the commensal strain PBIO365. Note that PBIO365 showed reduced growth in NB*sor* single culture. However, the advantage of PBIO729 was significantly diminished upon deletion of the *sorE* gene (*P* < 0.001). A similar depletion of competitive fitness was observed for PBIO729 (*P* < 0.001) and PBIO729∆*sorE* (*P* = 0.01) when competing against PBIO365:*sor* compared to the wild-type PBIO365. The observed competitive advantage of PBIO365:sor over PBIO729 demonstrates a relative fitness gain conferred by enabling L-sorbose utilization in an otherwise sorbose-negative ST10 background. In contrast, PBIO365 showed a reduced CI when competing against PBIO365:*sor*. These results highlight that the observed competitive advantage is dependent on the ability to utilize L-sorbose. We additionally observed that PBIO729 shows no competitive advantage over PBIO365 in the absence of L-sorbose (Fig. S8), suggesting that PBIO365 can rely on L-sorbose-independent mechanisms to compete effectively.

Consistent with the transcriptomic data, we detected high motility for PBIO729 (Fig. 3B). A slight but not significant increase in motility in the presence of L-sorbose was observed for PBIO729 and PBIO729∆*sorAM*, as well as for PBIO365 and PBIO365:*sor*. The PBIO729∆*sorE* mutant showed a significant reduction in motility in the presence of L-sorbose compared to the wild-type (*P* = 0.0057) and *sorAM* mutant (*P* = 0.0090). Interestingly, PBIO365 demonstrated a high level of motility, whereas PBIO365:*sor* exhibited a decrease in motility with considerable variation. Notably, we would not expect an increase in motility for PBIO365 in the presence of L-sorbose, as it cannot utilize L-sorbose, but the decreased motility observed in PBIO365:*sor* could reflect a complex interplay between L-sorbose utilization and motility regulation. This finding further emphasizes the importance of considering strain variability, as even small genetic differences can lead to considerable phenotypic divergence.

CPS plays a vital role as a virulence factor by, for example, protecting bacteria from components of the human immune system, such as those found in serum (45). Therefore, serum survival was assessed with L-sorbose and with D-glucose as a control. The commensal strain PBIO365 failed to survive under any condition (Fig. 3C and D). PBIO365: *sor* exhibited survival only in the presence of L-sorbose. The PBIO729Δ*sorE* mutant displayed significantly reduced serum survival in the presence of L-sorbose compared to the wild-type (*P* = 0.05) (Fig. 3C) and a notable reduction across all tested conditions compared to the wild-type strain and the PBIO729Δ*sorAM* mutant (Fig. 3D). The *Galleria mellonella in vivo* infection model was employed to evaluate the overall impact of the strains on fitness and potential virulence (Fig. 3E). Infection with PBIO365 resulted in moderate virulence, with survival rates of 80% during the early infection phase (≤48 h). By contrast, larvae infected with PBIO365:*sor* exhibited significantly lower survival rates, comparable to those infected with the highly virulent PBIO729, showing survival rates of only 20–30% after 48–72 h. Interestingly, the deletion of the *sorE* gene in PBIO729 did not affect larval mortality rates. It is important to note that L-sorbose could not be administered simultaneously during the infection experiments, and its occurrence needs to be investigated in future studies.

## DISCUSSION

Our study investigating 22,267 *E. coli* genomes revealed the *sor*-operon as a genomic marker enriched in pathogenic *E. coli* STs. The enrichment of the *sor-*operon in pathogenic strains across different phylogroups, in contrast to phylogroups A and B1, suggests a benefit for *sor*-carrying strains and its potential as a genetic marker for distinguishing between pathogenic and commensal *E. coli* isolates, though it does not serve as an absolute indicator of virulence. Our study offers a significant advancement over previous research with a comprehensive multi-omics approach including globally disseminated and clinically relevant MDR isolates from ST131 and ST648 (14). This research demonstrated the operon’s induction in the presence of L-sorbose. We highlight a link to increased energy production, consistent with the known functions of ribosomal proteins and ATPases in cellular physiology, and its importance for enhancing growth, although specific contributions in this context were not directly assessed. By not only enhancing growth but also driving the expression of virulence- and fitness-associated traits, this leads to competitive advantages for pathogenic *E. coli.* L-sorbose is a sugar present in fruits and detectable within different human and animal body fluids, e.g., feces and urine, but detailed studies on the concentration are lacking (46). Research in rats revealed fermentation of L-sorbose within the intestine after adaptation of the intestinal microflora (46). Furthermore, it is known that L-sorbose can be synthesized from D-sorbitol by *Acetobacter* sp (47), which resides in the gut of *Drosophila* sp., a commonly used model organism for human gut microbiome studies (48, 49). Importantly, sorbitol is a sugar alcohol commonly used as a sweetener in food and pharmaceutical products (50). The enhanced growth during the transient phase in complex media supports the idea that L-sorbose utilization promotes growth in nutrient-limited environments. Further, the findings suggest that strains capable of utilizing L-sorbose outcompete those lacking the *sor*-operon *in vitro*. In conclusion, L-sorbose utilization provides the potential to outcompete others in specific niches (51). For example, upon colonizing the intestine, *E. coli* must secure a nutrient niche, outcompeting other bacteria by dominating the use of at least one limiting nutrient (52). The genetic diversity of MDR *E. coli* in carbohydrate metabolism enhances their competitive edge over commensal *E. coli* in the intestinal tract of mice (53). Literature also highlights the importance of specific metabolic traits, such as amino acid and energy metabolism, for the fitness of pathogenic *E. coli* in mouse models (54, 55).

Upregulation of purine and tryptophan pathways indicates that pathogenic bacteria channel energy from L-sorbose fermentation into metabolic pathways associated with virulence. Tryptophan biosynthesis intermediates, such as indole, are known to modulate immune responses and affect gut barrier function (56)*.* The induction of L-sorbose (*sorC*) and purine metabolism (*purA*) has already been linked upon adhesion to human brain endothelial cells, with mutants showing reduced invasion (57). Moreover, UPEC relies on *de novo* purine synthesis to colonize purine-limited environments, such as the bladder, while mutations in purine biosynthesis (∆*purF*) impair colonization (58). L-sorbose is detectable in urine, suggesting that L-sorbose fermentation may enhance purine metabolism, boosting both bacteria’s fitness and virulence. Furthermore, PTSs can function in signal transduction in mediating the expression of virulence features (40). Comparable with the d fructose PTS, which mediates the expression of type 1 fimbriae in *E. coli*, supporting fitness and invasion into eukaryotic cells (59), we observed flagellar upregulation in the ST131 strain in the presence of L-sorbose. In addition, induction of capsule production alongside L-sorbose fermentation highlights the link between metabolic activities and virulence factor expression. Capsule synthesis and export serve as major virulence factors in many bacterial species, protecting the bacterium from the host immune response (60) and supporting colonization of murine kidneys and bladders (61). Although not statistically significant, the survival of PBIO365:*sor* and the killing of PBIO729∆*sorE* were specific to L-sorbose rather than glucose conditions, suggesting a direct link between L-sorbose utilization and serum survival.

Results from the *G. mellonella* infection model suggest that the *sor*-operon in the knock-in mutant of the commensal strain enhances virulence compared to the wild-type. In accordance with that, the involvement of L-sorbose utilization in pathogenesis has also been demonstrated for *Legionella pneumophila* (62). However, the absence of a significant effect from *sorE* deletion on mortality rates indicates that factors beyond L-sorbose metabolism may contribute to virulence. There is also a knowledge gap regarding the concentration and metabolism of L-sorbose *in vivo*, and whether the expression of the *sor*-operon is triggered. Future investigations in mouse models are planned to explore the competitive and fitness advantages conferred by L-sorbose utilization *in vivo*, especially in the presence of L-sorbose.

Overall, the regulatory profiles observed during L-sorbose fermentation underscore the critical relationship between metabolism and virulence. Previous studies have already shown that multidrug resistance alone does not explain the success of certain high-risk clonal lineages (63, 64). The link between enhanced metabolic pathways leading to increased virulence is well demonstrated, such as the association between aromatic amino acid biosynthesis and virulence in various bacterial species (65). Note that, while L-sorbose metabolism contributes to fitness and competition, its impact is not straightforward or universally applicable across all strains. Future studies should aim to explore how L-sorbose metabolism in other strains interacts with other metabolic pathways, virulence factors, and environmental conditions. Despite the observed complexities, the L-sorbose metabolism confers a competitive advantage beyond lL-sorbose utilization.

### Limitations

The study’s comparative genomic analysis was limited to a single commensal ST (ST10) due to the scarcity of publicly available genomes and metadata from other “commensal” *E. coli* lineages. However, non-pathogenic strains may acquire virulence traits over time. Another potential limitation is the inclusion of genomes from clonal populations in the homologous clustering. Due to limited metadata, filtering relied solely on strain names, but it did not significantly affect the overall results. Functional analyses were conducted using a limited number of strains, knock-out/knock-in mutants, and experiments. This may restrict the generalizability of the findings, particularly in *in vivo* experiments. Moreover, it is indispensable to perform studies to gain information about specific L-sorbose concentrations within different body sites to conclude about potential induction and related advantages of the *sor*-operon expression.

## Data Availability

The experimental and computational data supporting the findings of this research are available in this article and its supplementary information files. Genomic and transcriptomic data of this study have been deposited in the European Nucleotide Archive (ENA) at EMBL-EBI under accession number PRJEB98906. Proteome data have been stored at the ProteomeXchange Consortium via the PRIDE partner repository (34) with the dataset identifier PXD056542.

## SUPPLEMENTAL MATERIAL

Figure S1 msystems.01274-25-s0001.png Visualization of *sor* genomic region across STs.

Supplement msystems.01274-25-s0002.docx Supplemental methods, tables, and figures

Table S12 msystems.01274-25-s0003.xlsx List of genomes included in analysis of *sor* genomic region.

ASM does not own the copyrights to Supplemental Material that may be linked to, or accessed through, an article. The authors have granted ASM a non-exclusive, world-wide license to publish the Supplemental Material files. Please contact the corresponding author directly for reuse.
