# Supplementary figures and images for: Genomic and functional characterization of the L‑sorbose phosphotransferase system in high-risk Escherichia coli lineages
Source: mSystems. 2025 Dec 9;11(1):e01274-25. doi: 10.1128/msystems.01274-25 (PMC12817954; doi:10.1128/msystems.01274-25)

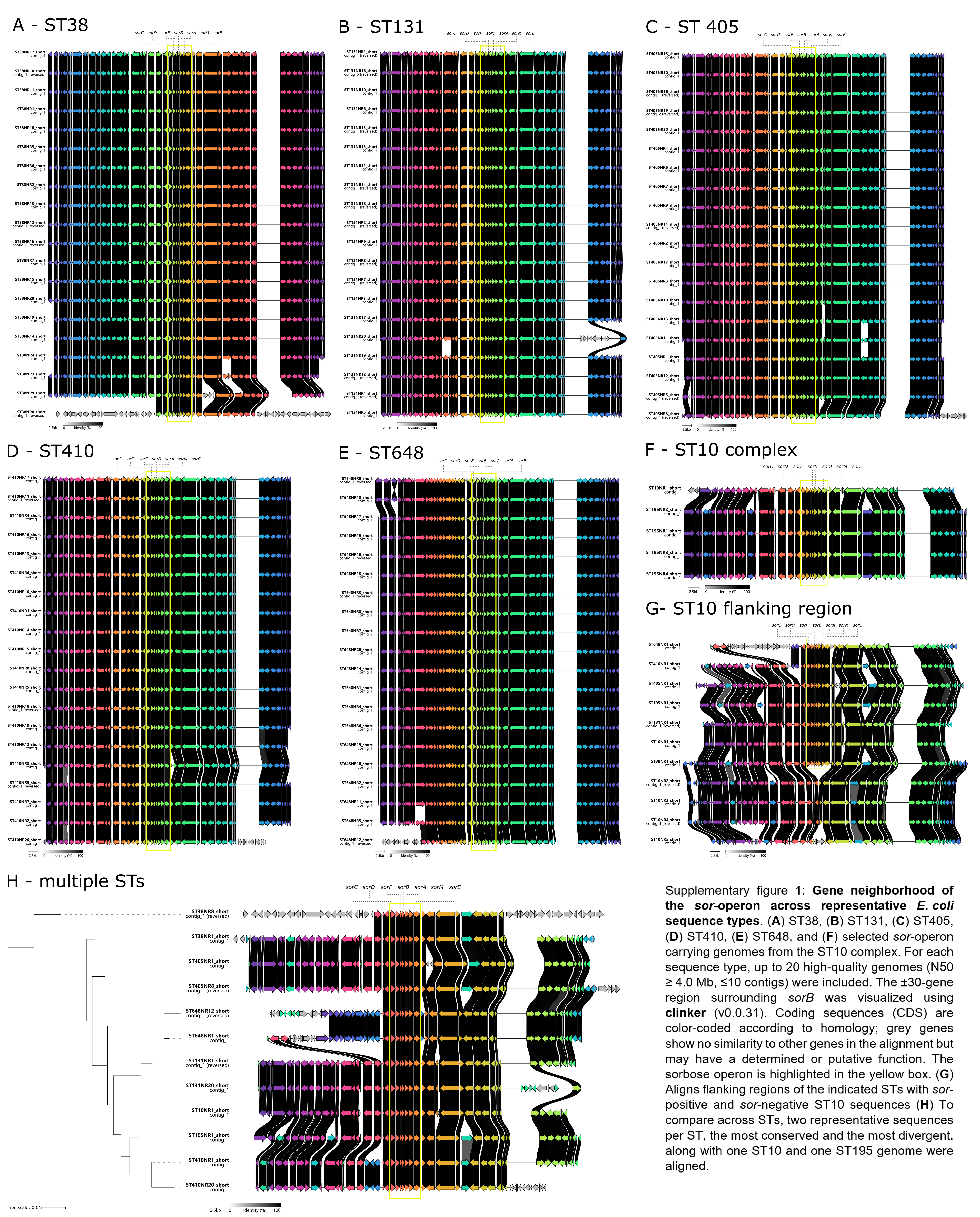

Supplement: Figure S1 — Visualization of sor genomic region across STs. [file msystems.01274-25-s0001.png]
