# Supplementary material for: Genomic and functional characterization of the L‑sorbose phosphotransferase system in high-risk Escherichia coli lineages
Source: mSystems. 2025 Dec 9;11(1):e01274-25. doi: 10.1128/msystems.01274-25 (PMC12817954; doi:10.1128/msystems.01274-25)
Supplement: Supplement — al methods, tables, and figures [file msystems.01274-25-s0002.docx]

## **The l‑sorbose** **phosphotransferase system: A virulence driver and potential therapeutic target in high-risk *Escherichia coli***

## **Supplementary methods**

### Mutant Generation and Sequencing

To verify mutant generation, total DNA was isolated and sent for sequencing. Library preparation was performed using the Illumina DNA Prep Kit and IDT 10 bp UDI indices (Illumina, San Diego, CA, USA). Short-read whole genome sequencing was carried out on an Illumina NextSeq 2000 (2 × 151 bp reads). Demultiplexing, quality control, and adapter trimming were performed using bcl-convert (v. 3.9.3) (1). Raw reads were trimmed with fastp (v. 0.23.4) (2) and were used as input for breseq (v. 0.38.1) (3) together with the respective parental genome annotation and were then manually evaluated. For the knock-in strain (PBIO365:*sor*) quality-trimmed reads were assembled with shovill.

### Northern blot analysis

Digoxigenin-labeled probes were used to hybridize with *sorC* or *sorE* transcripts. Probe templates were amplified from PBIO729 chromosomal DNA (**Supplementary table 3**) and synthesized via *in vitro* transcription (**Supplementary table 4**), as described previously by Mäder et al. in 2016. RNA samples were collected during mid-exponential, transient, and stationary growth phases of PBIO365, PBIO729, and PBIO730 cultivation in NB (ctrl and sor) (37 °C, 220 rpm). Denaturing agarose gel electrophoresis containing 1% agarose (Carl Roth), 2.1 M formaldehyde (Carl Roth) in 3-(*N*-morpholino)propanesulfonic acid (MOPS) buffer, and ethidium bromide loaded with 4 µg of purified RNA was performed. An in-house RNA sample buffer and RNA ladder were used. The membrane (Roche Diagnostics, Mannheim, Germany) was equilibrated with 20x saline sodium citrate (SSC) buffer. Denaturation and neutralization for 5 min each were performed prior to blotting (60 mBar for 3 h vacuum blot) with SSC transfer buffer. This was followed by UV crosslinking (Stratagene UVStratalinker 1800, La Jolla, CA, USA) and methylene blue staining. Upon pre-hybridization for one hour, probe hybridization was performed overnight at 68 °C using 1 µg each of the digoxigenin-labeled RNA probes. Subsequently, washing steps were conducted, 5 min with wash buffer 1 (room temperature (RT)) and three with wash buffer 2 (68 °C) for 15 min, then wash buffer 3 was added (5 min RT). The probe detection was achieved with an alkaline phosphatase-coupled anti-digoxigenin antibody (Anti-Digoxigenin-AP Fab; Roche, Basel, Switzerland) in blocking reagent for 30 min. Washing was repeated for 30 min and 60 min. The membrane was equilibrated for 5 min. Tropix CDP-*Star* (Thermo Fisher Scientific) was used as substrate (diluted 1:200 in equilibration buffer) for detection and documentation with Lumi Imager (Intas, Göttingen, Germany).

### Acquisition and Analysis of Mass Spectrometry Data

Database search was performed with parameters shown in **Supplementary table 6**. In brief, trypsin/P was set as the digesting enzyme with 2 missed cleavage allowed. Oxidation at methionine was set as variable modification. Peptides with min. seven amino acids displaying a q-value cut-off for detection of <0.001 were selected for further analyses. Only proteins detected with at least two peptides were considered for statistical analysis, which was performed using RStudio (v. 4.1.3 (2022-03-10)) with the tidyverse package (v. 1.3.2). Normalization factors were obtained from Spectronaut analysis. Missing values (intensity = 0) were replaced with the half-minimal intensity value from the whole dataset. Detected methionine oxidized peptides were excluded from further quantitative analysis. Ion intensities per sample and peptide were summed to calculate peptide intensities.

### Biolog plates

Biolog plates PM1, PM2, PM3, and PM5 (Biolog, Hayward, CA, USA) were used to screen the strains PBIO365, PBIO729, PBIO729∆*sorE*, and PBIO730 for their growth on various nutrients, with or without l-sorbose as an additional carbon source, to assess its impact on different metabolic pathways. The plates were prepared and processed following the manufacturer's protocol with slight modifications. Instead of using the dye, growth was monitored by measuring OD_600_. Growth differences were analyzed under both sorbose-supplemented and control conditions, as well as between wild-type and mutant strains. A pathway was considered influenced by sorbose if (a) it was unaffected in PBIO365, and (b) it was affected in PBIO729 and/or PBIO730. We focused on substrates that were unaffected in PBIO729∆*sorE* but affected in PBIO729, and evaluated these differences. To identify differences, the OD_600_ ratios were calculated, with ratios between 0.5 and 1.5 considered unaffected, and ratios below 0.5 or above 1.5 considered affected.

### Investigation of occurrence of the l-sorbose operon across phylogroups

We retrieved 44,208 E. coli genomes from RefSeq (taxid: 562, using NCBI Datasets command-line tool, v.18.3.1, 2 July 2025), classified with ezClermont (v0.7.0) and kleborate (v3.2.3), and screened for the sorbose operon using blastn (4, 5). Genomes containing all seven genes were considered positive. For each ST investigated in this study, a maximum of 20 high-quality genomes (N50 ≥ 4.0 Mb, ≤10 contigs) were randomly selected if possible, annotated with bakta (v1.11.3) (**Supplementary table 12**). To investigate the flanking regions, we extracted the ±30 locus ID region upstream and downstream of *sorB* and used clinker (v0.0.31) (6) for comparison and visualization of the CDS. We included ST195, as part of the ST10 complex, to increase the number of potential commensal strains in this analysis. Next we aligned (minimap2, v.2.30) all extracted up- and downstream (without the sor-genes) regions to 567 high-quality ST10 genomes to determine if these regions were also present in this sequence type. We selected 4 ST10 strains, which had different insert-length between the mapped regions for alignment and visualization with clinker (6).

## **Supplementary tables**

Supplementary table 1: **Overview of wild-type *E. coli* strains used in this study.** The strains included in all analyses are summarized and the phylogroups and number of genome assemblies used in the homologous clustering is shown. For STs that were further used for screenings and/or omics analysis, information on the representative isolate and its source and original description is provided.

| **ST** | **phylogroup** | **Number of included genomes** | **Strains included in-depth analysis** | **Original strain name** | **Host** | **Origin** | **Ref.** |
| --- | --- | --- | --- | --- | --- | --- | --- |
| ST10 | A | 8718 | PBIO2 | IMT 2312 | Chicken | Colibacillosis | (7) |
|  |  |  | PBIO32 | IMT 14960 | Companion animal | Genital tract infection | - |
|  |  |  | PBIO365* | IMT 35922 | Human | Rectal swab | (8) |
| ST38 | D | 1782 | PBIO879 | Mongolia 7766 | Wild bird  (crane) | Cloacal swab | - |
|  |  |  | PBIO876 | Mongolia 7763 | Wild bird  (crane) | Cloacal swab | - |
|  |  |  | PBIO3964 | 1 Eco04472 | Human | Freiburg Hospital | - |
| ST131 | B2 | 8923 | PBIO729* | IMT 17433 | Dog | UTI | (9) |
|  |  |  | PBIO1435 | IMT 18399 | Human | Healthy | - |
|  |  |  | PBIO1443 | IMT 27685 | Wild bird  (raven) | Feces | (9) |
| ST405 | D | 787 | PBIO241 | IMT 35147 | Human | Rectal swab | (8) |
|  |  |  | PBIO321 | IMT 35619 | Human | Rectal swab | (8) |
|  |  |  | PBIO3966 | 3 Eco04715 | Human | Freiburg Hospital | - |
| ST410 | C | 1044 | PBIO1633 | IMT 28707 | Wild bird  (Mute swan) | Cloacal swab | (10) |
|  |  |  | PBIO1643 | IMT 28764 | Wild bird  (Mute swan) | Cloacal swab | (10) |
|  |  |  | PBIO2963 | IMT 20031 | Unknown | Unknown | - |
| ST648 | F | 1013 | PBIO730 | IMT 16316 | Wild bird  (blackbird) | Feces | (9) |
|  |  |  | PBIO1434 | IMT 33167 | Unknown | Unknown | - |
|  |  |  | PBIO 1986 | IMT 17887 | Horse | Wound infection | (9) |

*additional mutant strains included: PBIO729∆*sorE*, PBIO729∆*sorAM* and PBIO365:*sor*

Supplementary table 2: **Composition of used buffers and solutions**

| **Method** | **Name** | **Composition** |
| --- | --- | --- |
| RNA preparation | **Killing buffer** | 20 mM Tris-HCl, pH 7.5; 5 mM MgCl_2_; 20 mM NaN_3_ |
| RNA preparation | **Lysis buffer** | 4 M guanidine-thiocyanate; 25 mM sodium acetate, pH 5.2; 0.5 % (wt/vol) sodium *N*-lauroylsarcosine |
| Northern Blot | **MOPS buffer** | 200 mM MOPS, Carl Roth; 50 mM sodium acetate, Merck KGaA; 10 mM EDTA Merck KGaA, pH 7.0 |
| Northern Blot | **RNA sample buffer** | 6.5 mL Formamide, Carl Roth; 1.2 mL Formaldehyde, Carl Roth; 2 mL 10x MOPS, 0.4ml 50% sucrose, Sigma Aldrich, Merck KGaA; 30 mg Orange G, Sigma Aldrich, Merck KGaA |
| Northern Blot | **SSC** | 300 mM tri-sodium citrate, Carl Roth; 3 M NaCl, Carl Roth; pH 7.0 |
| Northern Blot | **Denaturation** | 50 mM NaOH, Carl Roth; 10 mM NaCl, Carl Roth |
| Northern Blot | **Neutralization** | composition: 100 mM Tris-HCl, Merck KGaA; pH 7.4 |
| Northern Blot | **Methylene blue staining** | 100 µM methylene blue, Sigma Aldrich, Merck KGaA; 2% acetic acid, Carl Roth; 405 mM sodium acetate, Merck KGaA; pH 5.2 |
| Northern Blot | **Probe hybridization** | 0.02 % SDS, Merck KGaA; 0.1% n‑lauroylsarcosinate, Sigma Aldrich, Merck KGaA; 2 % Blocking reagent, Sigma Aldrich; Merck KGaA; 5 x SSC, 50% Formamide, Carl Roth |
| Northern Blot | **Wash buffer 1** | 2x SSC, 0.1 % SDS, Merck KGaA |
| Northern Blot | **Wash buffer 2** | 0.1x SSC, 0.1 % SDS, Merck KGaA |
| Northern Blot | **Wash buffer 3** | 0.3% Tween 20, Sigma Aldrich; Merck KGaA; 0.1 M Maleic acid, Carl Roth; 0.15 M NaCl, Carl Roth; 0.1925 M NaOH, Carl Roth |
| Northern Blot | **Blocking reagent** | 1% Blocking Reagent, 0.1 M Maleic acid, Carl Roth; 0.15 M NaCl, Carl Roth; 0.1925 M NaOH, Carl Roth |
| Northern Blot | **Equilibration buffer** | 100 mM Tris HCl, Merck KGaA, 100 mM NaCl, Carl Roth; 1 M diethanolamine, Carl Roth; pH 9.5. |

Supplementary table 3: PCR reaction and program for preparation of the probe templates

| **PCR reaction mix** | | | | | | |
| --- | --- | --- | --- | --- | --- | --- |
| 70 µL | nuclease free dH_2_O | | (in-house) | | |  |
| 20 µL | HF-Buffer | | (Thermo Fisher Scientific, Waltham, MA, USA) | | |  |
| 2 µL | MgCl_2_ | | (Thermo Fisher Scientific, Waltham, MA, USA) | | |  |
| 2 µL | dNTPs [10 mM] | | (Thermo Fisher Scientific, Waltham, MA, USA) | | |  |
| 2 µL | Primer 1 [100 pM] | | (Eurofins Genomics, Ebersberg, Germany) | | |  |
| 2 µL | Primer 2 [100 pM] | | Eurofins Genomics, Ebersberg, Germany) | | |  |
| 2 µL | Template | | (DNA of PBIO729 [62 ng/µL]) | | |  |
| 0.5 µL | Phusion polymerase | | (Thermo Fisher Scientific, Waltham, MA, USA) | | |  |
| **PCR program** | | | | | | |
| Initial denaturation | | 98°C | | 2 min |  | |
| Denaturation | | 98°C | | 30 s | 30 cycles | |
| Annealing | | 66°C – 63°C – 60°C (10 cycles each) | | 30 s |  |  |
| Extension | | 72°C | | 20 s |  |  |
| Final Extension | | 72°C | | 2 min |  | |
| Hold | | 6°C | | forever |  | |
| **Agarose gel electrophoresis** | | | | | | |
| 1 % agarose gel | | |  |  |  | |
| 0.01% ethidium bromide | | |  |  |  | |
| 100 bp GeneRuler | | | 160 V |  |  | |

Supplementary table 4: Reaction mix and protocol for *in vitro* transcription of digoxigenin probes

| **Reaction mix for *in-vitro* transcription for digoxigenin probes** | | | | |
| --- | --- | --- | --- | --- |
| 0.5 µg | cleaned PCR product | NucleoSpin, Gel and PCR Clean-up (Macherey&Nagel) | | |
| 2 µL | 10x reaction buffer | DIG RNA Labeling Kit (SP6/T7) (Roche) | | |
| 2 µL | NTP mix | DIG RNA Labeling Kit (SP6/T7) (Roche) | | |
| 1 µL | RNase inhibitor | RNasin® Ribonuclease Inhibitor (Promega). | | |
| 2 µL | T7-RNA Polymerase | DIG RNA Labeling Kit (SP6/T7) (Roche) | | |
| ad. 20 µL | H_2_O |  | | |
| **Reaction protocol** | | | | |
| Incubation | | | 37°C | 2 h |
| 2 µL DNase I | | | 37°C | 15 min |
| 2 µL 0.2 M EDTA (pH8) | | |  |  |
| 2.5 µL 4 M LiCl | | |  |  |
| 75 µL 96% EtOH | | |  |  |
| Precipitation | | | -20°C | Overnight |
| Centrifugation | | | 4°C | 15 min |
| Wash pellet | | | 1 mL | 70% EtOH |
| Centrifugation | | | 4°C | 15 min |
| Dry pellet | | |  |  |
| Resolve pellet in 100 µL *A. dest* + 1µL RNA inhibitor | | | 37°C | 30 min |
| Store | | | -80°C |  |

Supplementary table 5: **Instrumental setting for reversed phase liquid chromatography (RPLC) and mass spectrometry.**

| Reversed phase liquid chromatography (RPLC) | |
| --- | --- |
| Instrument | Ultimate 3000 RSLC (Thermo Scientific) |
| Trap column | 75 μm inner diameter, packed with 3 μm C18 particles (Acclaim PepMap100, Thermo Scientific) |
| Analytical column | Accucore 150-C18, (Thermo Fisher Scientific)  25 cm x 75 μm, 2.6 μm C18 particles, 150 Å pore size |
| Buffer system | binary buffer system consisting of 0.1% acetic acid in HPLC-grade water (buffer A) and 100% ACN in 0.1% acetic acid (buffer B) |
| Flow rate | 300 nl/min |
| Gradient | 0 min 2% B 🡪  2 min 5% B 🡪  10 min 5% B 🡪  130 min 25% B 🡪  135 min 40% B 🡪  137 min 90% B 🡪  142 min 90% B 🡪  145 min 2% B 🡪  150 min 2% B |
| Gradient duration | 120 min |
| Column oven temperature | 40°C |
| **Mass spectrometry** | |
| Instrument | Q Exactive HF mass spectrometer |
| Electrospray | Nanospray Flex Ion Source |
| Operation mode | data-independent |
| Full MS |  |
| MS scan resolution | 60000 |
| Norm. AGC target | 5e6 |
| maximum ion injection time for the MS scan | 200 ms |
| Scan range | 333 to 1650 m/z |
| RF Lens | 50% |
| Spectra data type | profile |
| dd-MS2 |  |
| Precursor mass range | 333 to 1650 m/z |
| Resolution | 30,000 |
| Norm. MS/MS AGC target | 3e6 |
| Maximum ion injection time mode | auto |
| Spectra data type | profile |
| Microscans | 1 |
| Isolation window | 56 windows, 13 m/z, 2 m/z overlap |
| Define first mass | 200 |
| Dissociation mode | higher energy collisional dissociation (HCD) |
| Normalized collision energy | 27.5 % |

Supplementary table 6: **Spectronaut settings for mass spectrometry.**

| ANALYSIS DATA |
| --- |
| Spectronaut 16.0.220606.53000  Analysis Type: directDIA |
| Settings Used: C_FunGene_directDIA_sparse_no_imputing |
| ├─ DIA Analysis\Calibration  │ ├─ MZ Extraction Strategy: Maximum Intensity  │ ├─ Allow source specific iRT Calibration: True  │ ├─ Precision iRT: True  │ │ ├─ Exclude De-amidated Peptides: True  │ │ └─ iRT <-> RT Regression Type: Local (Non-Linear) Regression  │ ├─ MS1 Mass Tolerance Strategy: System Default  │ └─ MS2 Mass Tolerance Strategy: System Default  ├─ DIA Analysis\Identification  │ ├─ Precursor Qvalue Cutoff: 0.001  │ ├─ Precursor PEP Cutoff: 0.2  │ ├─ Protein Qvalue Cutoff (Experiment): 0.01  │ ├─ Protein Qvalue Cutoff (Run): 0.05  │ ├─ Single Hit Definition: By Stripped Sequence  │ ├─ Exclude Single Hit Proteins: False  │ ├─ Exclude Duplicate Assays: True  │ ├─ Generate Decoys: True  │ │ ├─ Decoy Generation Method: Mutated  │ │ │ └─ Preferred Fragment Source: NN Predicted Fragments  │ │ └─ Decoy Limit Strategy: Dynamic  │ │ └─ Library Size Fraction: 0.1  │ └─ Pvalue Estimator: Kernel Density Estimator  ├─ DIA Analysis\Pipeline Mode  │ ├─ Generate SNE File: True  │ │ └─ Store Ion traces in SNE: False  │ ├─ Post Analysis Reports:  │ │ ├─ CV Density Line Chart: True  │ │ ├─ CVs Below X Bar Chart: True  │ │ ├─ Data Completeness Bar Chart: True  │ │ ├─ Run Identifications Bar Chart: True  │ │ └─ Scoring Histograms: True  │ ├─ Report Schema: C_FunGene_complex (Normal)  │ └─ Reporting Unit: Across Experiment  ├─ DIA Analysis\Post Analysis  │ ├─ Differential Abundance Testing: Paired t-test  │ │ └─ Group-Wise Testing Correction: False  │ ├─ Differential Abundance Grouping: Major Group (Quantification Settings)  │ │ └─ Smallest Quantitative Unit: Precursor Ion (Quantification Settings)  │ │ └─ Use All MS-Level Quantities: False  │ ├─ Calculate Explained TIC: Quick  │ ├─ Calculate Sample Correlation Matrix: True  │ └─ Hierarchical Clustering: True  │ ├─ Distance Metric: Manhattan Distance  │ ├─ Linkage Strategy: Ward's Method  │ ├─ Order Runs by Clustering: True  │ └─ Z-score Transformation: False  ├─ DIA Analysis\Protein Inference  │ └─ Protein Inference Workflow: Automatic  │ └─ Inference Algorithm: IDPicker  ├─ DIA Analysis\PTM Workflow  │ └─ PTM Localization: True  │ ├─ Probability Cutoff: 0.75  │ └─ PTM Analysis: True  │ ├─ Hierarchical Clustering: False  │ ├─ Multiplicity: True  │ ├─ Flanking Region: 7  │ └─ PTM Consolidation: Sum  ├─ DIA Analysis\Quantification  │ ├─ Precursor Filtering: Identified (Qvalue)  │ │ └─ Imputation Strategy: Use Background Signal  │ ├─ Proteotypicity Filter: None  │ ├─ Protein LFQ Method: Automatic  │ ├─ Quantity MS Level: MS2  │ ├─ Quantity Type: Area  │ ├─ Cross-Run Normalization: True  │ │ ├─ Normalization Filter Type: None  │ │ ├─ Normalization Strategy: Local Normalization  │ │ └─ Row Selection: Identified in at least 1 Run (Sparse)  │ ├─ Interference Correction: True  │ │ ├─ Only Identified Peptides: True  │ │ ├─ Exclude All Multi-Channel Interferences: True  │ │ ├─ MS1 Min: 2  │ │ └─ MS2 Min: 3  │ ├─ Major (Protein) Grouping: by Protein Group Id  │ ├─ Minor (Peptide) Grouping: by Stripped Sequence  │ ├─ Major Group Quantity: Mean peptide quantity  │ ├─ Major Group Top N: True  │ │ ├─ Max: 3  │ │ └─ Min: 2  │ ├─ Minor Group Quantity: Sum precursor quantity  │ └─ Minor Group Top N: False  ├─ DIA Analysis\Workflow  │ ├─ Method Evaluation: False  │ ├─ MS2 DeMultiplexing: Automatic  │ ├─ Profiling Strategy: iRT Profiling  │ │ ├─ Carry-over exact Peak Boundaries: False  │ │ ├─ Profiling Row Selection: Minimum Qvalue Row Selection  │ │ │ └─ Qvalue Threshold: 0.001  │ │ └─ Profiling Target Selection: Profile only non-identified Precursors  │ │ ├─ Identification Criterion: Qvalue  │ │ └─ Threshold: 0.001  │ ├─ Run Limit for directDIA Library: -1  │ └─ Unify Peptide Peaks Strategy: Select corresponding Peak  ├─ DIA Analysis\XIC Extraction  │ ├─ XIC IM Extraction Window: Dynamic  │ │ └─ Correction Factor: 1  │ ├─ XIC RT Extraction Window: Dynamic  │ │ └─ Correction Factor: 1  │ ├─ MS1 Mass Tolerance Strategy: Dynamic  │ │ └─ Correction Factor: 1  │ └─ MS2 Mass Tolerance Strategy: Dynamic  │ └─ Correction Factor: 1  ├─ Pulsar Search\Identification  │ ├─ PSM FDR: 0.01  │ ├─ Peptide FDR: 0.01  │ ├─ Protein Group FDR: 0.01  │ └─ PTM Localization Filter: False  ├─ Pulsar Search\Labeling  │ └─ Channels:  │ ├─ Channel 1: False  │ ├─ Channel 2: False  │ └─ Channel 3: False  ├─ Pulsar Search\Modifications  │ ├─ Max Variable Modifications: 5  │ └─ Select Modifications:  │ ├─ Fixed Modifications::  │ └─ Variable Modifications: : Oxidation (M)  ├─ Pulsar Search\Peptides  │ ├─ Enzymes / Cleavage Rules: Trypsin/P  │ ├─ Digest Type: Specific  │ ├─ Max Peptide Length: 52  │ ├─ Min Peptide Length: 7  │ ├─ Missed Cleavages: 2  │ └─ Toggle N-terminal M: True  ├─ Pulsar Search\Result Filters  │ ├─ Fragment Ions:  │ │ ├─ Ion AA Length: True  │ │ │ └─ N: 3  │ │ ├─ Ion Charge: False  │ │ ├─ Ion Loss Type: False  │ │ ├─ Ion Type: False  │ │ ├─ m/z : True  │ │ │ ├─ Max: 1800  │ │ │ └─ Min: 300  │ │ └─ Relative Intensity: True  │ │ └─ Min: 5  │ └─ Precursors:  │ ├─ Amino Acids: False  │ ├─ Best N Fragments per Peptide: True  │ │ ├─ Max: 10  │ │ └─ Min: 6  │ ├─ Best N Peptides per Protein Group: False  │ ├─ Channel Count: False  │ ├─ FASTA Matched: False  │ ├─ Missed Cleavage: False  │ ├─ Modifications: None  │ ├─ Peptide Charge: False  │ └─ Proteotypicity: False  ├─ Pulsar Search\Speed-Up  │ └─ MS2 Index: Automatic  ├─ Pulsar Search\Tolerances  │ └─ Tolerance Parameters:  │ ├─ Thermo IonTrap:  │ │ ├─ Calibration Search: Dynamic  │ │ │ ├─ MS1 Correction Factor: 1  │ │ │ └─ MS2 Correction Factor: 1  │ │ └─ Main Search: Dynamic  │ │ ├─ MS1 Correction Factor: 1  │ │ └─ MS2 Correction Factor: 1  │ ├─ Thermo Orbitrap:  │ │ ├─ Calibration Search: Dynamic  │ │ │ ├─ MS1 Correction Factor: 1  │ │ │ └─ MS2 Correction Factor: 1  │ │ └─ Main Search: Dynamic  │ │ ├─ MS1 Correction Factor: 1  │ │ └─ MS2 Correction Factor: 1  │ └─ TOF:  │ ├─ Calibration Search: Dynamic  │ │ ├─ MS1 Correction Factor: 1  │ │ └─ MS2 Correction Factor: 1  │ └─ Main Search: Dynamic  │ ├─ MS1 Correction Factor: 1  │ └─ MS2 Correction Factor: 1  └─ Pulsar Search\Workflow  ├─ Fragment Ion Selection Strategy: Intensity Based  ├─ In-Silico Generate Missing Channels: False  └─ Use DNN Predicted Ion Mobility: Auto |

Supplementary table 7: **Colony PCR**

| **PCR reaction mix** | | | | | | | |
| --- | --- | --- | --- | --- | --- | --- | --- |
| 6.25 µL | DreamTaq Green PCR Master Mix (2x) | | (Thermo Fisher Scientific, Waltham, MA, USA) | | | |  |
| 0.5 µL | Primer 1 [100 pM] (AGGAGGCTTTATGGTCAATGC) | | (Eurofins Genomics, Ebersberg, Germany) | | | |  |
| 0.5 µL | Primer 2 [100 pM]  (CCAGATCGCTGCGTGTAA) | | Eurofins Genomics, Ebersberg, Germany) | | | |  |
| 2.75 µL | dH_2_O | |  | | | |  |
| 2.5 µL | Template | | (colony resuspended in 10 µl dH_2_O and cooked 5 min at 95°C) | | | | |
| **PCR program** | | | | | | | |
| Initial denaturation | | 95°C | | | 3 min |  | |
| Denaturation | | 95°C | | | 30 s | 25 cycles | |
| Annealing | | 51°C | | | 30 s |  |  |
| Extension | | 72°C | | | 1 min |  |  |
| Final Extension | | 72°C | | | 5 min |  | |
| Hold | | 8°C | | | forever |  | |
| **Agarose gel electrophoresis** | | | | | | | |
| 1 % agarose gel | | |  | |  |  | |
| 1x GelRed® Nucleic Acid Stain (Merck KGaA) | | | |  |  |  | |
| 100 bp GeneRuler | | | 160 V | |  |  | |

Supplementary table 8: **Genomic markers**: Percentage of occurrence of *sor-*operon genes within the analyzed *E. coli* STs.

| ST10 | ST38 | ST131 | ST405 | ST410 | ST648 | annotation (Sp hit) |
| --- | --- | --- | --- | --- | --- | --- |
| 0.069 | 99.944 | 99.765 | 99.746 | 99.617 | 98.322 | sp\|P37084\|SORE_KLEPN L-sorbose 1-phosphate reductase OS=Klebsiella pneumoniae OX=573 GN=sorE PE=3 SV=1 |
| 0.057 | 99.607 | 99.675 | 99.365 | 99.33 | 97.335 | sp\|P37078\|SORC_KLEPN Sorbitol operon regulator OS=Klebsiella pneumoniae OX=573 GN=sorC PE=1 SV=1 |
| 0.069 | 99.944 | 99.832 | 99.873 | 99.521 | 99.309 | sp\|P37083\|PTRD_KLEPN PTS system sorbose-specific EIID component OS=Klebsiella pneumoniae OX=573 GN=sorM PE=2 SV=1 |
| 0.069 | 99.888 | 99.731 | 99.619 | 99.713 | 99.901 | sp\|P37082\|PTRC_KLEPN PTS system sorbose-specific EIIC component OS=Klebsiella pneumoniae OX=573 GN=sorA PE=2 SV=1 |
| 0.057 | 100 | 99.832 | 99.492 | 99.617 | 98.815 | sp\|P37079\|SORD_KLEPN Sorbitol-6-phosphate 2-dehydrogenase OS=Klebsiella pneumoniae OX=573 GN=sorD PE=3 SV=1 |
| 0.069 | 100 | 99.675 | 99.873 | 99.521 | 99.803 | sp\|P37081\|PTRB_KLEPN PTS system sorbose-specific EIIB component OS=Klebsiella pneumoniae OX=573 GN=sorB PE=1 SV=1 |

Supplementary table 9: **Occurrence of genes of the l-sorbose operon across phylogroups**: The percentage of genomes carrying the l‑sorbose (sorbose-positive) are shown. The analysis included a total of 44,208 *E. coli* genomes from RefSeq, whereas 248 remained unclassified by EzClermont.

| Phylogroup | Sorbose-positive (%) | Number of genomes |
| --- | --- | --- |
| A | 9.59% | 12654 |
| B1 | 12.25% | 12243 |
| B2 | 96.98% | 8090 |
| C | 96.10% | 1822 |
| D | 86.94% | 4382 |
| E | 88.81% | 2413 |
| F | 95.45% | 1450 |
| G | 90.82% | 730 |
| U | 59.19% | 174 |

Supplementary table 10: **Regulations shared on transcriptomic and proteomic levels:** The L2FC is shown for the comparison of sorbose to sorbose free conditions for the indicated strains on transcriptomic (trans) and proteomic (prot) level. Group numbers, descripton of protein product and gene names are shown. List contains all regulations that differ significantly for either one or both pathogenic strains (PBIO729 and PBIO730) but not for the commensal counterpart (PBIO365).

| Group | Description | gene | trans PBIO365 | trans PBIO729 | trans PBIO730 | prot PBIO365 | prot PBIO729 | prot PBIO730 |
| --- | --- | --- | --- | --- | --- | --- | --- | --- |
| g06078 | Shikimate dehydrogenase-like protein HI_0607 | *sdhL* | 0.00 | 0.00 | 6.01 | 0.02 | -0.11 | 6.77 |
| g06101 | PTS system sorbose-specific EIIC component | *sorA* | 0.00 | 7.30 | 5.67 | 0.36 | 12.67 | 13.02 |
| g03021 | PTS system sorbose-specific EIIA component | *sorF* | 0.00 | 7.40 | 5.67 | -0.20 | 5.42 | 2.86 |
| g04456 | PTS system sorbose-specific EIIB component | *sorB* | 0.00 | 7.37 | 5.65 | 0.10 | 9.44 | 4.60 |
| g03971 | L-sorbose 1-phosphate reductase | *sorE* | 0.00 | 7.10 | 5.62 | -0.13 | 2.62 | 0.58 |
| g03711 | Sorbitol-6-phosphate 2-dehydrogenase | *sorD* | 0.00 | 7.42 | 5.60 | 0.58 | 11.23 | 8.51 |
| g03685 | PTS system sorbose-specific EIID component | *sorM* | 0.00 | 7.36 | 5.58 | -0.67 | 9.36 | 5.29 |
| g03535 | Sorbitol operon regulator | *sorC* | 0.00 | 6.89 | 5.55 | -0.23 | 7.29 | 5.79 |
| g00934 | PTS system glucitol/sorbitol-specific EIIB component | *srlE* | 0.00 | 4.10 | 4.23 | -0.03 | 1.91 | 2.46 |
| g05856 | Arabinose 5-phosphate isomerase KpsF | *kdsD* | 0.00 | 0.00 | 1.86 | 0.36 | -0.26 | 3.30 |
| g01389 | Maltodextrin phosphorylase | *malP* | 0.00 | 0.00 | 1.53 | 0.06 | -0.25 | 1.01 |
| g06232 | Sucrose porin | *scrY* | 0.00 | 6.02 | 0.00 | -0.01 | 8.52 | -0.28 |
| g05924 | Fructokinase | *scrK* | 0.00 | 5.51 | 0.00 | -0.45 | 6.99 | 0.14 |
| g06304 | PTS system sucrose-specific EIIBC component | *treP* | 0.00 | 5.27 | 0.00 | 0.16 | 4.63 | -1.18 |
| g01650 | Sulfate/thiosulfate import ATP-binding protein CysA | *cysA* | 0.00 | 3.51 | 0.00 | 0.35 | 1.89 | 0.17 |
| g06282 | Sucrose-6-phosphate hydrolase | *scrB* | 0.00 | 3.40 | 0.00 | -0.10 | 4.95 | -0.30 |
| g01956 | 2-methylisocitrate lyase | *prpB* | 0.00 | 3.28 | 0.00 | 0.28 | -1.97 | 0.03 |
| g02306 | 2-methylcitrate dehydratase | *prpD* | 0.00 | 3.14 | 0.00 | -0.33 | -2.12 | -0.41 |
| g02595 | 2-methylcitrate synthase | *prpC* | 0.00 | 3.11 | 0.00 | -0.05 | -2.43 | -0.23 |
| g01506 | Propionate--CoA ligase | *prpE* | 0.00 | 2.74 | 0.00 | 0.01 | -2.20 | -0.13 |
| g05774 | Flagellin | *fliC* | 0.00 | 1.66 | 0.00 | 0.03 | -1.56 | 0.58 |
| g05576 | Putative uncharacterized protein YaiT | *-* | 0.00 | 1.49 | 0.00 | 0.13 | -0.36 | -1.59 |
| g02410 | Maltoporin | *lamB* | 0.00 | 1.31 | 0.00 | -0.18 | -1.54 | -0.01 |
| g06201 | Type I restriction enzyme StySPI M protein | *-* | 0.00 | 1.24 | 0.00 | -0.21 | 0.18 | -1.36 |
| g05920 | hypothetical_protein_1 | *-* | 0.00 | 1.18 | 0.00 | -0.13 | -2.58 | -0.03 |
| g02168 | Methyl-accepting chemotaxis protein I | *tsr* | 0.00 | 1.12 | 0.00 | -0.57 | -1.91 | -0.41 |
| g00814 | Flagellar brake protein YcgR | *ycgR* | 0.00 | 1.01 | 0.00 | -0.84 | -2.14 | -0.31 |
| g01800 | D-xylose-binding periplasmic protein | *xylF* | 0.00 | -1.18 | 0.00 | -0.08 | -1.67 | 0.04 |
| g02209 | Uncharacterized protein YdhS | *ydhS* | 0.00 | -1.25 | 0.00 | -0.37 | -1.82 | -0.55 |
| g02106 | Lysine/arginine/ornithine-binding periplasmic protein | *argT* | 0.00 | -1.28 | 0.00 | -0.33 | -1.35 | -0.03 |
| g06322 | Long-chain fatty acid transport protein | *fadL* | 0.00 | -1.33 | 0.00 | 0.24 | -1.41 | 1.03 |
| g01545 | Long-chain-fatty-acid--CoA ligase | *fadD* | 0.00 | -1.44 | 0.00 | -0.53 | -1.32 | -0.81 |
| g02272 | Putrescine aminotransferase | *patA* | 0.00 | -1.50 | 0.00 | -0.78 | -1.76 | -1.24 |
| g03379 | Putrescine transport ATP-binding protein PotG | *potG* | 0.00 | -1.53 | 0.00 | -0.86 | -1.22 | -0.54 |
| g02435 | sn-glycerol-3-phosphate-binding periplasmic protein UgpB | *ugpB* | 0.00 | -1.53 | 0.00 | -0.49 | -1.29 | -0.56 |
| g01633 | Putrescine-binding periplasmic protein PotF | *potF* | 0.00 | -1.54 | 0.00 | -0.77 | -1.09 | -0.47 |
| g01428 | Fatty acid oxidation complex subunit alpha | *fadB* | 0.00 | -1.55 | 0.00 | -0.35 | -1.27 | -0.46 |
| g01467 | 2,4-dienoyl-CoA reductase [(2E)-enoyl-CoA-producing] | *fadH* | 0.00 | -1.57 | 0.00 | -0.51 | -1.02 | -0.38 |
| g03487 | L-arabinose-binding periplasmic protein | *araF* | 0.00 | -1.90 | 0.00 | -0.47 | -1.97 | -0.09 |
| g02408 | N-succinylarginine dihydrolase | *astB* | 0.00 | -2.04 | 0.00 | -0.78 | -1.44 | -0.79 |
| g01742 | Arginine N-succinyltransferase | *astA* | 0.00 | -2.05 | 0.00 | -0.73 | -1.49 | -0.97 |
| g01598 | Bifunctional polyhydroxybutyrate synthase / ABC transporter periplasmic binding protein | *ydcS* | 0.00 | -2.06 | 0.00 | -0.29 | -1.33 | -0.14 |
| g01824 | Glutarate 2-hydroxylase | *csiD* | 0.00 | -1.91 | -1.45 | -0.19 | -1.98 | -0.36 |
| g00325 | Cyclopropane-fatty-acyl-phospholipid synthase | *cfa* | 0.00 | -1.55 | -1.19 | -0.66 | -1.43 | -0.95 |

Supplementary table 11: **Biolog screening:** Biolog plates PM1, PM2, PM3, and PM5 were used to screen the strains PBIO365, PBIO729, PBIO729∆*sorE*, and PBIO730 for their growth on various nutrients, with or without L-sorbose as an additional carbon source. Growth was assessed my measuring OD_600_ before and after 24 h incubation. Substances showing differences between conditions without (ctrl) and with sorbose (sor) are shown. For that the OD600 ratios were calculated, with ratios between 0.5 and 1.5 considered unaffected, and ratios below 0.5 or above 1.5 considered affected.

| Substance | PBIO365 ctrl | PBIO365 sor | PBIO730 ctrl | PBIO730 sor | PBIO729 ctrl | PBIO729 sor | PBIO729 ∆*sorE* ctrl | PBIO729 ∆*sorE* sor |
| --- | --- | --- | --- | --- | --- | --- | --- | --- |
| 2,3-Butanediol | 0.949 | 0.648 | 0.933 | 0.35 | 0.419 | 0.685 | 0.794 | 0.752 |
| Phenylethylamine | 0.896 | 0.727 | 0.626 | 1.069 | 0.267 | 0.9 | 0.663 | 0.691 |
| 2-Aminoethanol | 1.03 | 0.687 | 0.48 | 0.563 | 0.337 | 0.653 | 0.702 | 0.616 |
| DL-Octopamine | 0.665 | 0.655 | 0.825 | 0.556 | 0.361 | 0.66 | 0.67 | 0.458 |
| L-Serine | 1.004 | 1.017 | 1.044 | 1.227 | 0.519 | 1.061 | 0.777 | 0.653 |
| L-Isoleucine | 0.826 | 0.638 | 0.292 | 0.57 | 0.33 | 0.929 | 0.54 | 0.649 |
| L-Leucine | 0.82 | 0.699 | 0.69 | 0.688 | 0.636 | 1.034 | 0.813 | 0.73 |
| L-Lysine | 0.704 | 0.7 | 0.778 | 0.538 | 0.463 | 0.924 | 0.756 | 0.691 |
| L-Methionine | 0.906 | 0.633 | 0.646 | 0.46 | 0.439 | 0.762 | 0.772 | 0.768 |
| Fumaric Acid | 1.151 | 1.158 | 1.24 | 1.073 | 0.605 | 1.051 | 1.004 | 0.757 |
| Glyoxylic Acid | 1.248 | 1.178 | 1.239 | 1.393 | 0.77 | 1.345 | 0.969 | 0.855 |
| D-Malic Acid | 1.454 | 1.134 | 1.329 | 1.304 | 0.521 | 1.251 | 0.588 | 0.579 |
| L-Tartaric Acid | 0.807 | 0.728 | 0.897 | 0.47 | 0.513 | 1.106 | 0.715 | 0.562 |
| D-Cellobiose | 0.877 | 0.636 | 0.833 | 0.803 | 0.353 | 1.168 | 0.954 | 0.698 |
| Bromo Succinic Acid | 0.982 | 1.086 | 1.394 | 1.35 | 0.553 | 1.147 | 0.851 | 0.773 |
| Guanosine | 0.844 | 0.726 | 0.518 | 0.608 | 0.188 | 0.397 | 0.447 | 0.54 |
| Glycyl-L-Aspartic Acid | 0.85 | 1.113 | 1.042 | 1.282 | 0.729 | 1.137 | 0.765 | 0.677 |
| Glycyl-L-Glutamic Acid | 0.987 | 1.04 | 0.608 | 1.143 | 0.604 | 1.109 | 0.478 | 0.653 |
| Propionic Acid | 1.271 | 0.938 | 0.693 | 1.193 | 0.49 | 0.838 | 0.698 | 0.737 |
| Tween 80 | 0.456 | 0.627 | 0.911 | 1.064 | 0.325 | 1.247 | 0.554 | 0.58 |

Supplementary table 12 is provided as an extra file named Supplementary_table_12.xlsx: Supplementary table 12: **Genome name and Kleborate results for the 105 genomes for in-depth characterization of the *sor*-operon surrounding regions**: The table contains the genome information, including accession number, ST, phylogroup, clonal_complex, species, contig_count, N50 assigned using Kleborate (v.3.2.3), ezClermont (v.0.7.0).

## **Supplementary figures**


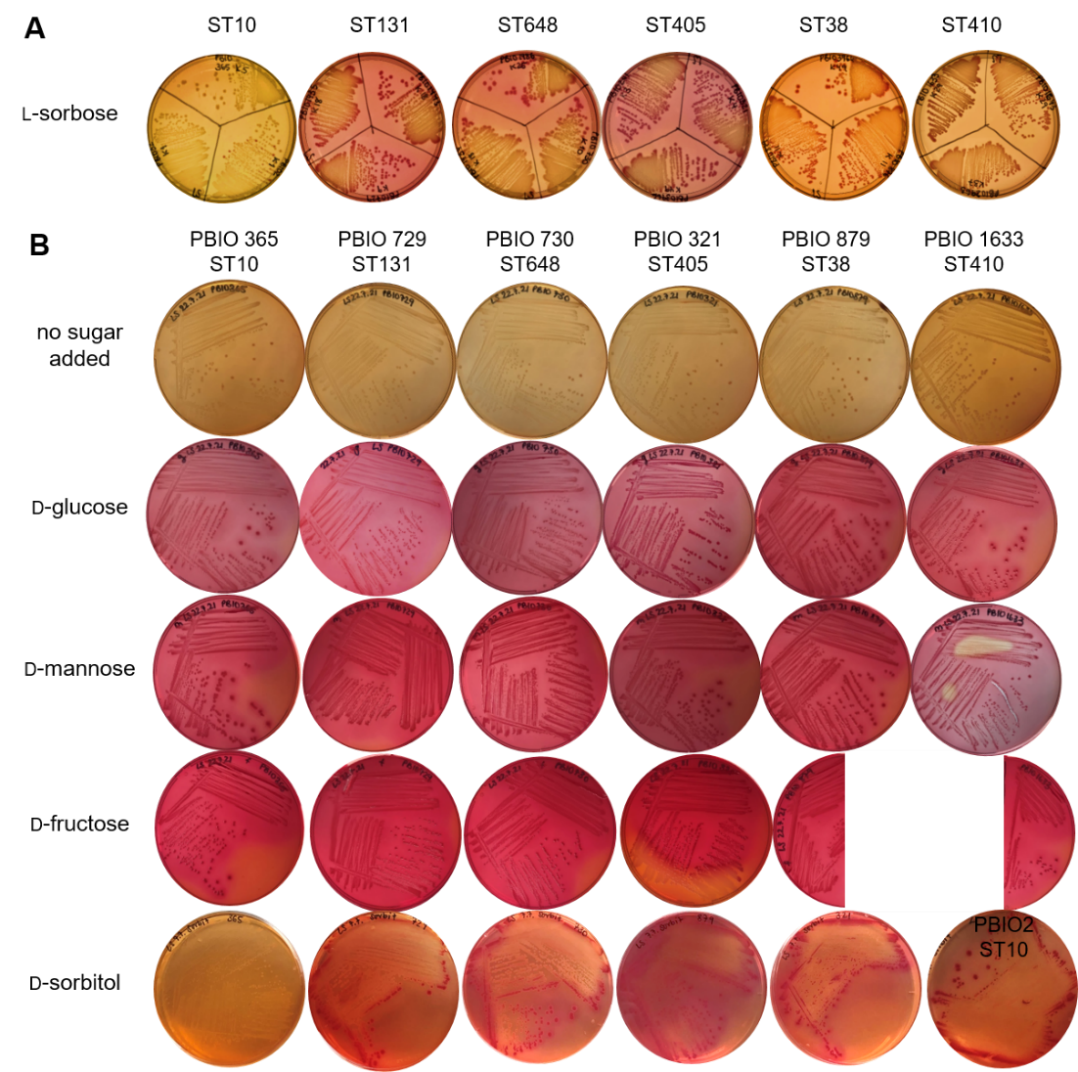


Supplementary figure 2: **Testing utilization patterns of different *E. coli* strains for various sugars:** 10 g/l of each sugar as well as 24 mg/l neutral red were added to Nutrient Broth II agar. Indicated *E. coli* strains were streaked out from glycerol stocks at incubated for 16 h at 37 °C. (**A**) Three strains of indicated ST were tested for l-sorbose utilization. (**B**) Each of the indicated sugars was tested for utilization by a representative strain of each ST.


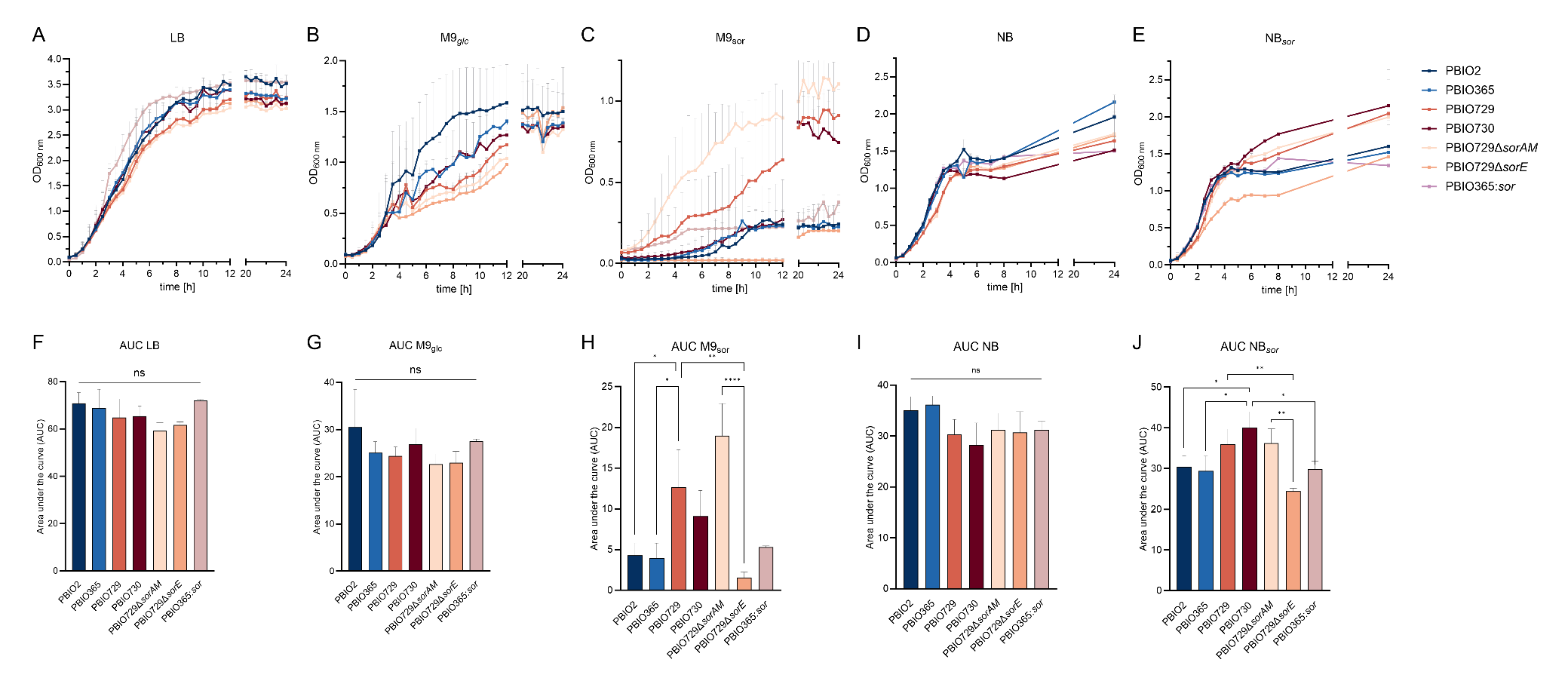


Supplementary figure 3: **Growth kinetics and calculation of area under the curves from cultivation in various media:** Indicated *E. coli* strains were cultivated in (**A**) LB, (**B**) M9 minimal medium containing 2 mM MgSO_4_ and 1% d-glucose, (**C**) M9 minimal medium containing 2 mM MgSO_4_ and 1% l-sorbose, (**D**) NB, (**E**) NB supplemented with 1% l-sorbose. Calculation of the area under the curve (**F-J**) was performed using GraphPad, as well as statistical analysis (One-Way ANOVA) from three independent biological replicates (adj. *p* > 0.05 = not significant (ns), < 0.033 (*); < 0.002 (**); < 0.001 (***)).


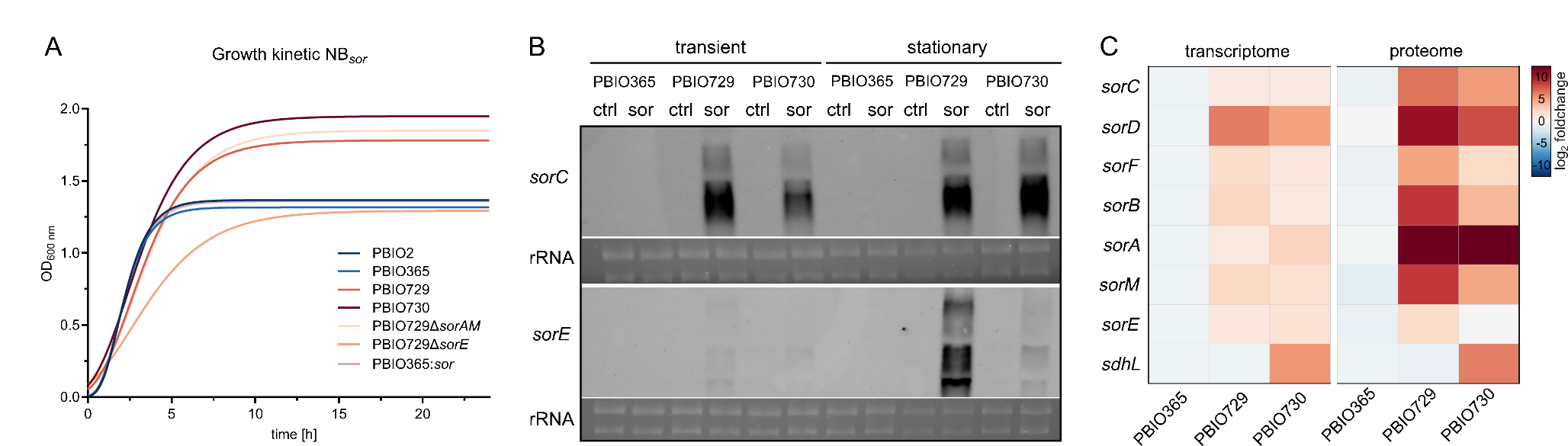


Supplementary figure 4: **Initiation of l-sorbose utilization**: (**A**) Growth kinetics of pathogenic and commensal *E. coli* STs in the presence and absence of l-sorbose: Wild-type pathogenic (PBIO729 and PBIO730), mutant strains (PBIO729∆*sorAM* but not PBIO729∆*sorE*) and commensals (PBIO365 and PBIO2) were cultivated in NB supplemented with 1% l-sorbose. The OD_600 nm_ was determined regularly to measure growth in three biological replicates. Growth curves were modulated using Gompertz growth fitting in GraphPad (**B**) Detection of l‑sorbose PTS mRNA by Northern Blotting using digoxigenin-labeled probes against *sorC* and *sorE*: RNA samples (4 µg) isolated from the cultivation of one commensal (PBIO365) and two pathogenic (PBIO729 and PBIO730) strains in the absence (ctrl) or presence of 1% l‑sorbose (sor). The samples were taken during transient growth phase and stationary growth phase (transient + 2 h). Loading control of the ethidium bromide-stained agarose gel is shown below. Blotting was performed for 3 h at 60 mbar. An in-house size RNA ladder served a size standard. Detection was performed by addition of an anti-digoxigenin antibody to which an alkaline phosphatase is coupled. CDP-Star served as substrate of the chemoluminescence reaction. (**C**) Heatmap of DGE observed comparing l-sorbose and control samples on transcriptomic and proteomic levels: The DGE between samples isolated from strains (PBIO365, PBIO729 and PBIO730) grown in presence of 1% l‑sorbose compared to those grown in the absence of l-sorbose is expressed as L2FC for data from transcriptome and proteome analyses, respectively. Only significant values are displayed (|L2FC|≥1; adj. *p* < 0.05), otherwise values are displayed as 0.


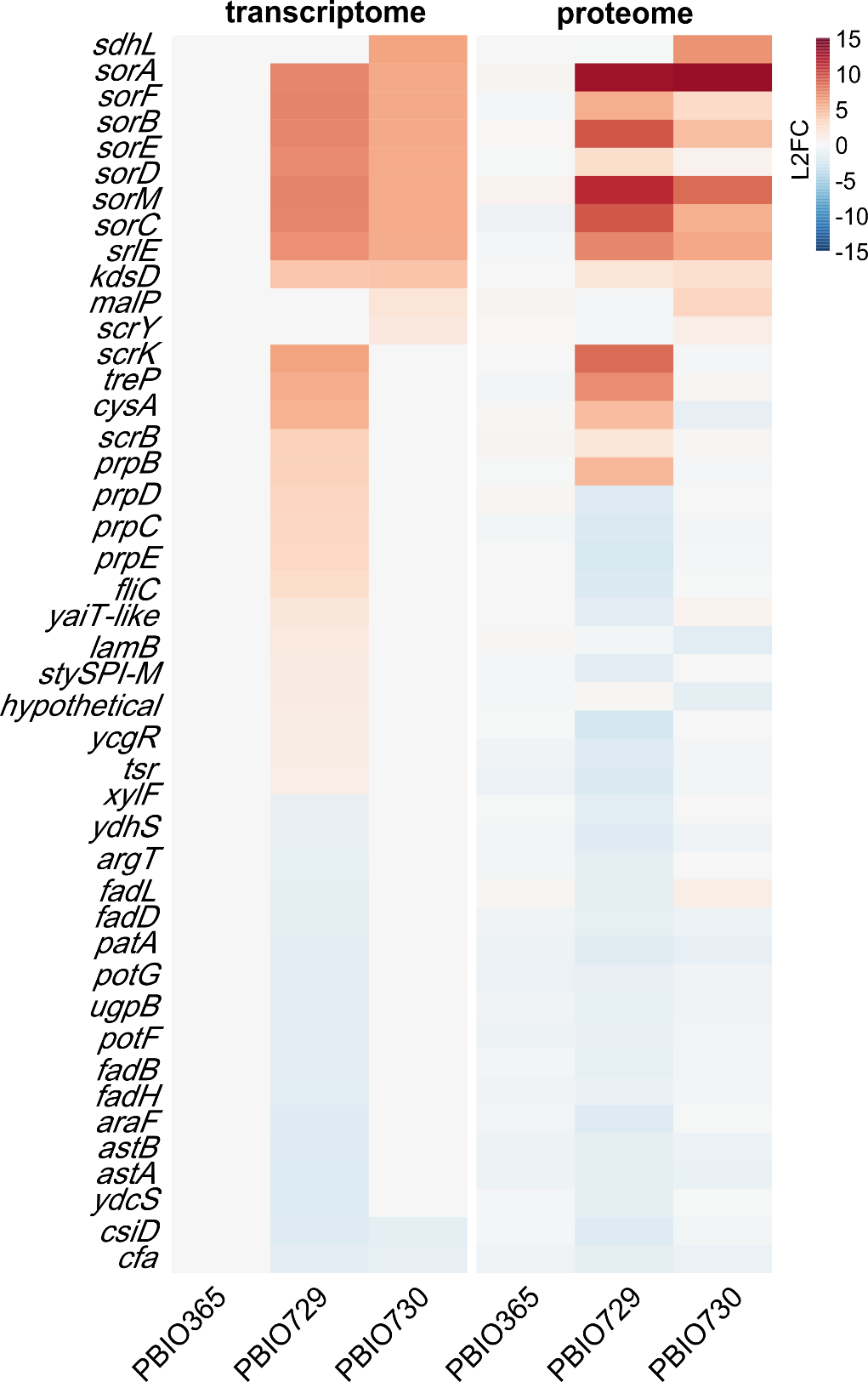


Supplementary figure 5: **Heatmap of the L2FC of transcripts and protein of shared on transcriptomic and proteomic levels:** The heatmap of L2FC was calculated comparing l-sorbose and control samples on transcriptomic and proteomic levels. Gene names are shown. List contains all regulations that differ significantly for either one or both pathogenic strains (PBIO729 and PBIO730) but not for the commensal counterpart (PBIO365) on transcriptomic and proteomic level.


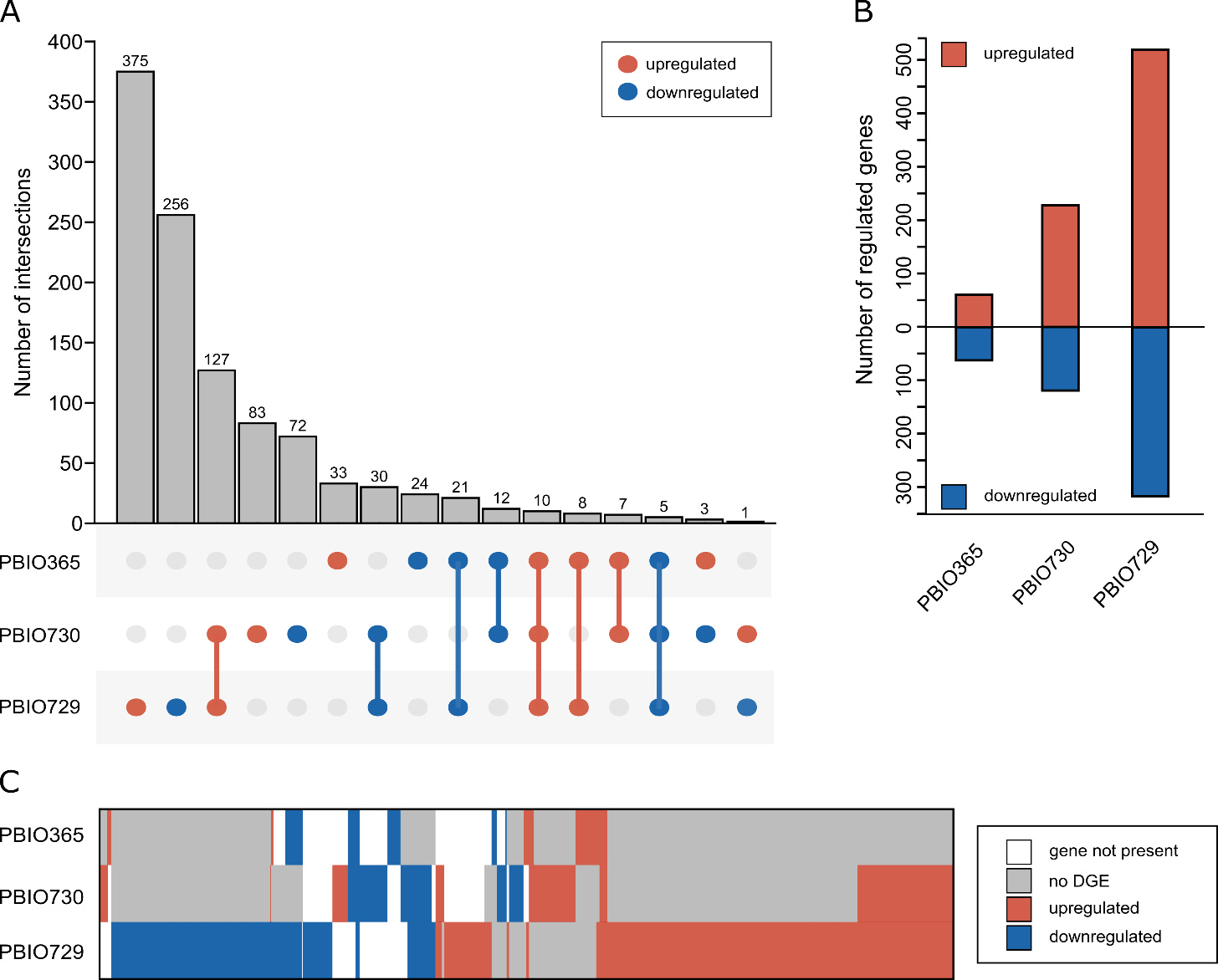


Supplementary figure 6: **Visualization of regulations on transcriptomic level upon cultivation in the presence of l‑sorbose:** (**A**) Upset plot visualizing shared genes differentially expressed on transcriptomic level: Bars are ranked by frequency representing the total numbers of genes uniquely up- (red) and/or downregulated (blue) (|L2FC| ≥1.0; *p* adjusted ≤ 0.05) in the respective combination. (**B**): Total number of up- (red) or downregulated (blue) genes plotted for each strain. (**C**) Heatmap of regulated transcripts: Regulated genes (|L2FC| ≥1.0; *p* adjusted ≤ 0.05) under sor compared to ctrl conditions are displayed for each strain distinguishing between genes that are not present in the genome (white), genes not differentially expressed (grey), upregulated (red), and downregulated genes (blue).


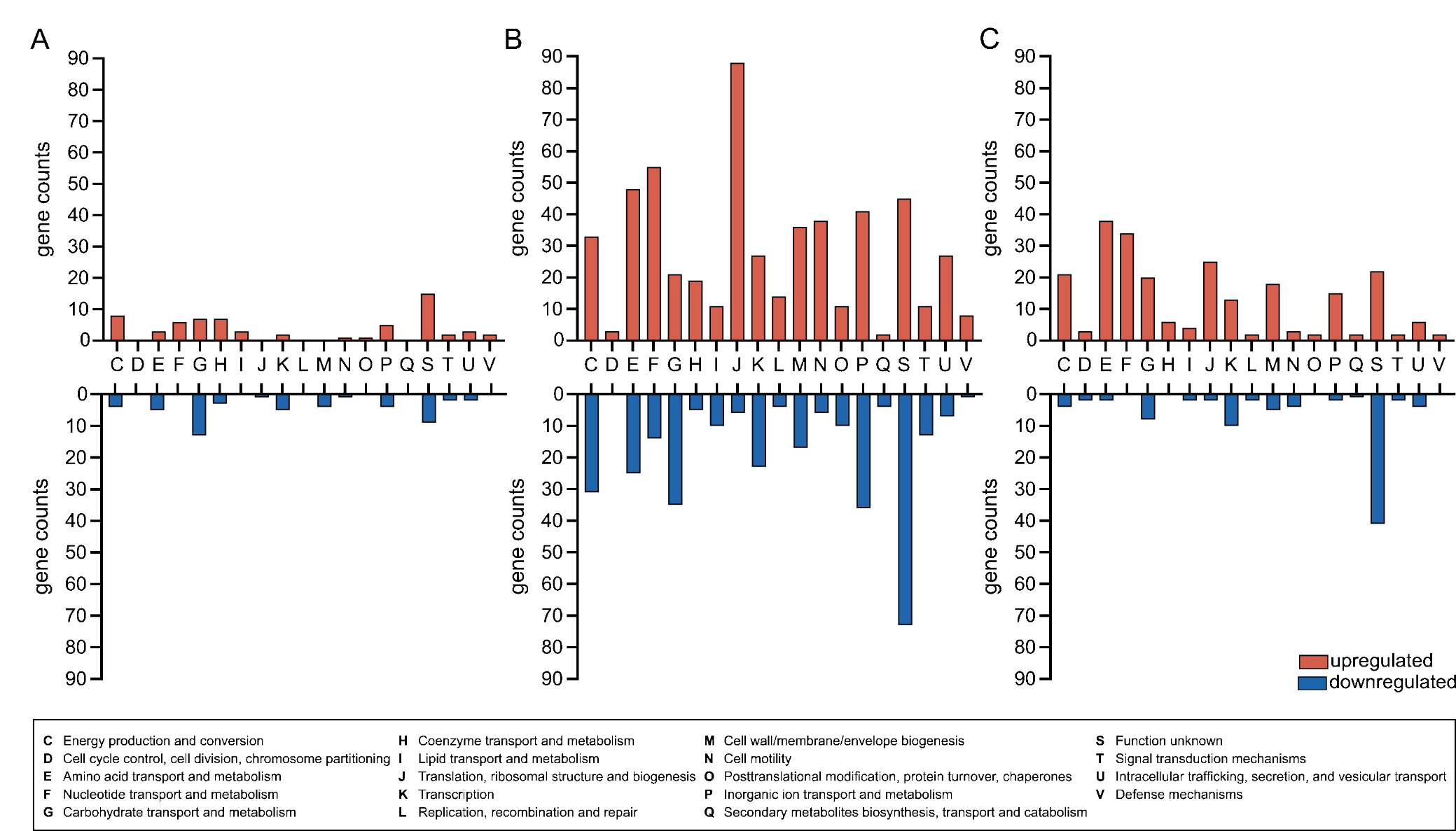


Supplementary figure 7: **Visualization of counts of up- or downregulated genes belonging to the same COG category**: The number of differentially expressed genes (|L2FC| ≥ 1.0; p-adjusted ≤ 0.05) belonging to one COG category were plotted for (**A**) ST10 (PBIO365) (**B**) ST131 (PBIO729) and (**C**) ST648 (PBIO730). Regulations are differentiated between upregulated (red) and downregulated (blue). Explanations of each COG category are shown.


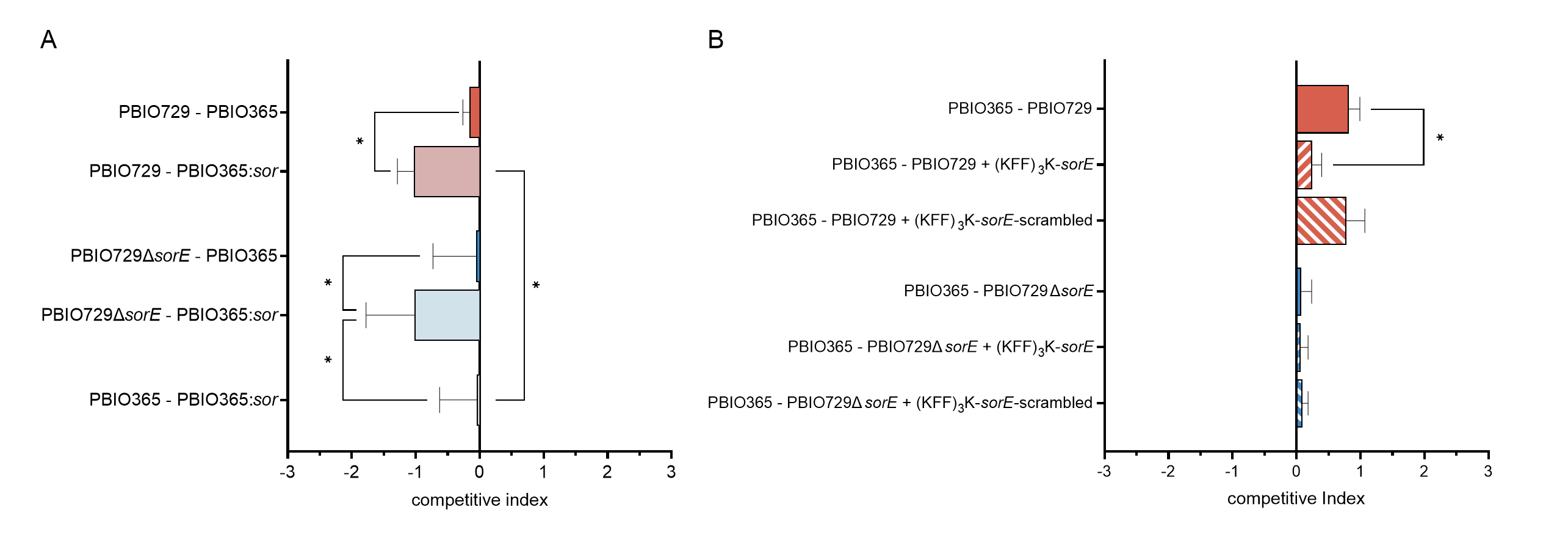


Supplementary figure 8: **Competition assay in Nutrient Broth II without l-sorbose**: The *E. coli* ST10 representative PBIO365, the pathogenic wild-type strain (PBIO729), its *sorE-*mutant (PBIO729∆*sorE*) and the PBIO365:*sor* strain were tested in biological replicates (n=3) for (**A**) competitive benefits in Nutrient Broth medium. The CFU/ml of the competing strains was calculated by plating on non-selective and selective LB-agar and/or analyzed by colony PCR. The competitive index was calculated comparing the ratio of test over reference strain after 24 h of competition with the inoculation ratio. Relevant changes were evaluated regarding their significance (ordinary One-Way ANOVA with Fisher’s LSD test; adj. *p* < 0.033 (*); < 0.002 (**); < 0.0002 (***); < 0.0001 (****)).

## References

1. Prjibelski A, Antipov D, Meleshko D, Lapidus A, Korobeynikov A. 2020. Using SPAdes De Novo Assembler. Curr Protoc Bioinforma 70.

2. Chen S. 2023. Ultrafast one-pass FASTQ data preprocessing, quality control, and deduplication using fastp. iMeta 2:1–5.

3. Deatherage DE, Barrick JE. 2014. Identification of mutations in laboratory evolved microbes from next-generation sequencing data using breseq Daniel. Methods Enzymol 1151:165–188.

4. Waters NR, Abram F, Brennan F, Holmes A, Pritchard L. 2020. Easy phylotyping of Escherichia coli via the EzClermont web app and command-line tool. Access Microbiol 2.

5. Lam MMC, Wick RR, Watts SC, Cerdeira LT, Wyres KL, Holt KE. 2021. A genomic surveillance framework and genotyping tool for Klebsiella pneumoniae and its related species complex. Nat Commun 12.

6. Gilchrist CLM, Chooi YH. 2021. Clinker & clustermap.js: Automatic generation of gene cluster comparison figures. Bioinformatics 37:2473–2475.

7. Martinez-Medina M, Garcia-Gil J, Barnich N, Wieler LH, Ewers C. 2011. Adherent-Invasive Escherichia coli Phenotype Displayed by Intestinal Pathogenic E . coli Strains from Cats , Dogs , and Swine ᰔ. Appl Environ Microbiol 77:5813–5817.

8. Eger E, Heiden SE, Korolew K, Bayingana C, Ndoli JM, Sendegeya A, Gahutu JB, Kurz MSE, Mockenhaupt FP, Müller J, Simm S, Schaufler K. 2021. Circulation of Extended-Spectrum Escherichia coli of Pandemic Among Hospitalized Patients, Caregivers, and the Community in Rwanda. Front Microbiol 12:1–11.

9. Schaufler K, Semmler T, Pickard DJ, De Toro M, De La Cruz F, Wieler LH, Ewers C, Guenther S. 2016. Carriage of extended-spectrum beta-lactamase-plasmids does not reduce fitness but enhances virulence in some strains of pandemic *E. coli* lineages. Front Microbiol 7.

10. Schaufler K, Semmler T, Wieler LH, Wöhrmann M, Baddam R, Ahmed N, Müller K, Kola A, Fruth A, Ewers C, Guenther S. 2016. Clonal spread and interspecies transmission of clinically relevant ESBL-producing Escherichia coli of ST410-another successful pandemic clone? FEMS Microbiol Ecol 92:1–9.
